# Bioengineering the metabolic network of CAR T cells with GLP-1 and Urolithin A increases persistence and long-term anti-tumor activity

**DOI:** 10.1016/j.xcrm.2025.102021

**Published:** 2025-03-18

**Authors:** Areej Akhtar, Md Shakir, Mohammad Sufyan Ansari, Divya, Md Imam Faizan, Varnit Chauhan, Aashi Singh, Ruquaiya Alam, Iqbal Azmi, Sheetal Sharma, Mehak Pracha, Insha Mohi Uddin, Uzma Bashir, Syeda Najidah Shahni, Rituparna Chaudhuri, Sarah Albogami, Rik Ganguly, Shakti Sagar, Vijay Pal Singh, Gaurav Kharya, Amit Kumar Srivastava, Ulaganathan Mabalirajan, Soumya Sinha Roy, Irfan Rahman, Tanveer Ahmad

**Affiliations:** 1 Multidisciplinary Centre for Advanced Research and Studies, Jamia Millia Islamia, New Delhi, India; 2 Indian Institute of Science, Centre for Brain Research, Bengaluru, Karnataka, India; 3 Department of Biotechnology, College of Science, Taif University, Taif, Saudi Arabia; 4 Department of Biotechnology & Bioinformatics, North-Eastern Hill University, Shillong, India; 5 CSIR-Institute of Genomics & Integrative Biology, New Delhi, India; 6 Centre for Bone Marrow Transplant & Cellular Therapy, Indraprastha Apollo Hospital, New Delhi, India; 7 CSIR-Indian Institute of Chemical Biology, Kolkata, West Bengal, India; 8 Department of Environmental Medicine, University of Rochester Medical Center, Rochester, NY, USA

**Keywords:** CAR T cells, metabolism, GLP-1 peptide, Urolithin A, autophagy, mitophagy, mitochondrial health, T cell persistence, anti-tumor activity

## Abstract

Constant tumor antigen exposure disrupts chimeric antigen receptor (CAR) T cell metabolism, limiting their persistence and anti-tumor efficacy. To address this, we develop metabolically reprogrammed CAR (MCAR) T cells with enhanced autophagy and mitophagy. A compound screening identifies a synergy between GLP-1R agonist (semaglutide [SG]) and Urolithin A (UrA), which activate autophagy through mTOR (mechanistic target of rapamycin) inhibition and mitophagy via Atg4b activation, maintaining mitochondrial metabolism in CAR T cells (MCAR T-1). These changes increase CD8^+^ T memory cells (Tm), enhancing persistence and anti-tumor activity *in vitro* and in xenograft models. GLP-1R knockdown in CAR T cells diminishes autophagy/mitophagy induction, confirming its critical role. We further engineer GLP-1-secreting cells (MCAR T-2), which exhibited sustained memory, stemness, and long-term persistence, even under tumor re-challenge. MCAR T-2 cells also reduce cytokine release syndrome (CRS) risks while demonstrating potent anti-tumor effects. This strategy highlights the potential of metabolic reprogramming via targeting autophagy/mitophagy pathways to improve CAR T cell therapy outcomes, ensuring durability and efficacy.

## Highlights

• TR triggers CAR T cell decline, which is reversed by GLP-1RA/SG and UrA (MCAR T cells)
• MCAR T cells synergistically rejuvenate function via autophagy/mitophagy
• MCAR T cells ensure long-term persistence and cytotoxicity in pre-clinical models

## Graphical abstract

**Figure:**
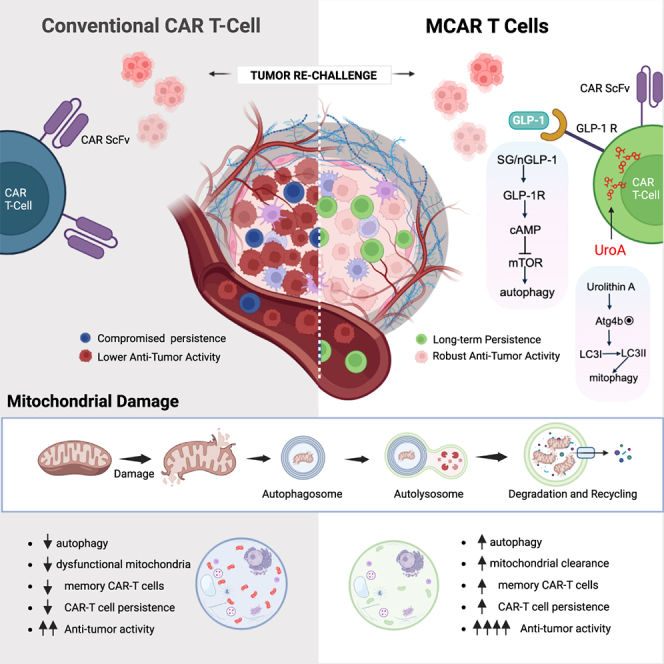

## Teaser

Akhtar et al. show that bioengineering CAR T cells with GLP-1R agonist and Urolithin A enhances autophagy/mitophagy, leading to improved persistence and anti-tumor activity. This approach offers a promising strategy to overcome CAR T cell exhaustion and improve therapeutic efficacy.

## Introduction

Chimeric antigen receptor (CAR) T cell therapy has revolutionized cancer treatment, demonstrating remarkable efficacy in B cell malignancies.^1^ CD8^+^ cytotoxic effector T (T_eff_) cells are essential for initiating primary adaptive immune responses.^2^^,^^3^ Following antigen clearance, T_eff_ cells can transition into memory T (T_m_) cells, forming a long-lasting subset capable of rapid secondary immune responses upon antigen re-exposure.^4^^,^^5^ However, chronic antigen stimulation often induces T cell exhaustion.^6^^,^^7^ Among T_eff_ and T_m_ cells, subsets like T effector memory (T_em_) and T central memory (T_cm_) exhibit increased persistence and differentiation potential,^7^ highlighting their importance in anti-tumor immunity. Understanding how metabolic programs regulate T cell functionality across states is critical for optimizing CAR T cell therapies.

Emerging evidence emphasizes the role of mitochondrial metabolism in shaping T cell behavior, influencing epigenetic features and functionality.^8^^,^^9^^,^^10^^,^^11^^,^^12^ While T_eff_ cells rely on glycolysis and one-carbon metabolism,^11^ T_m_ cells adopt a distinct metabolic profile characterized by increased fatty acid oxidation (FAO) and mitochondrial spare respiratory capacity (SRC), supporting long-term survival. This metabolic shift is vital for memory and stem-like properties. However, how T cells adapt mitochondrial metabolism during chronic antigen stimulation and the impact on their functions remain unclear. High-quality mitochondria are essential for T cell activation, differentiation, and tumor-fighting ability.^13^^,^^14^^,^^15^^,^^16^

Mitochondrial biogenesis, structure, and dynamics, particularly fusion in T_m_ and fission in T_eff_, play key roles in T cell function.^17^^,^^18^ Fusion proteins like Opa1 enhance T_m_ cell oxidative phosphorylation (OXPHOS) and FAO by remodeling mitochondria, while T_eff_ cells prioritize glycolysis due to cristae expansion and reduced electron transport chain (ETC) efficiency.^17^^,^^19^ Recent studies on CAR T cells emphasize the metabolic impact of co-receptor domains like CD28 and 4-1BB. CD28 supports glycolysis in effector memory cells, while 4-1BB enhances T_cm_ persistence, FAO, and mitochondrial biogenesis.^20^^,^^21^ These findings emphasize the role of mitochondrial metabolism and quality control in CAR T cell efficacy.

Autophagy, especially mitophagy, is critical for clearing damaged mitochondria and maintaining T cell functionality. It plays a key role in memory formation, stemness, and metabolic activity.^22^^,^^23^^,^^24^ Despite the increased autophagy observed in proliferating T cells, its role in CAR T cells is not fully understood.^25^^,^^26^ Knockout models reveal that impaired autophagy disrupts T cell homeostasis and development.^27^^,^^28^ However, the specific link between autophagy, mitophagy, and long-term persistence, especially in CAR T cells, remains elusive. To address these challenges, this study demonstrates that metabolic reprogramming of CAR T cells can be achieved by targeting autophagy and mitophagy pathways. By screening a small library of molecules involved in these processes and mitochondrial metabolism, we identified GLP-1RA/semaglutide (SG) and Urolithin A (UrA) as promising candidates for enhancing CAR T cell persistence and anti-tumor activity. These molecules promote autophagy and mitophagy, thereby improving CAR T cell metabolic activity, long-term persistence, and memory formation.

## Results

### Tumor re-challenge causes CAR T cell dysfunction due to impaired mitophagy and decrease in autophagosome formation

**Figure 1:**
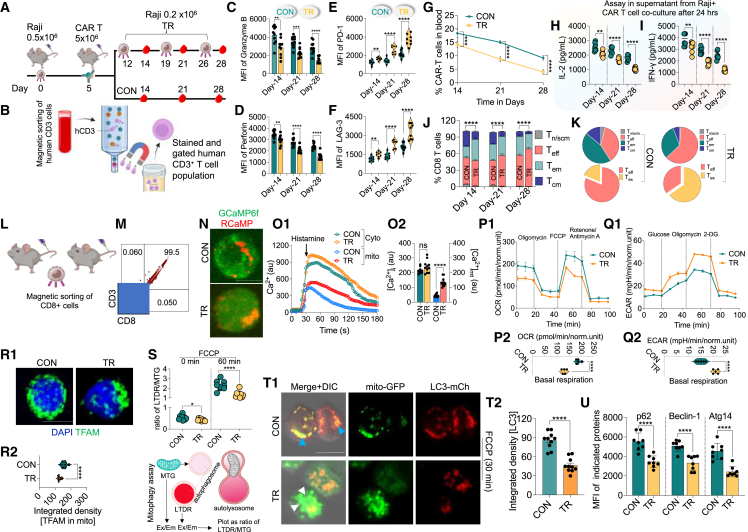
Functional and metabolic characterization of CAR T cells after repeated tumor re-challenge (A) Schematic of the lymphoma model. Mice were injected with Raji cells (0.5 × 10^6^) on day 0, followed by CAR T cell (5 × 10^6^) administration on day 5. TR was done on days 12, 19, and 26 with 0.2 × 10^6^ Raji cells. Blood was collected on days 14, 21, and 28. (B) Sorting and flow cytometry gating of human CD3^+^ CAR T cells from blood samples across time points (3 mice were pooled for a total of 24 in each group to obtain *n* = 8 per condition). (C–F) Flow cytometric analysis showing expression of Granzyme B (C), Perforin (D), PD-1 (E), and LAG-3 (F) in CON and TR groups over time (*n* = 8; mean ± SD). The data are represented as mean fluorescence intensity (MFI) calculated from the flow cytometry histograms. (G–I) Percentage of CAR T cells in blood tracked over time (G). IL-2 (H) and IFN-γ (I) secretion quantified from co-culture of CAR T cells with Raji cells for 24 h (*n* = 8). (J and K) Human CD8^+^ T cell subsets (T_n_, T_scm_, T_cm_, T_em_, and T_eff_) in CD3^+^ T cells measured by flow cytometry in TR vs. CON groups at each time point. The pie chart shows the percentage of T_ex_ in the T_eff_ population. (L and M) Sorting of human CD8^+^ cells from pooled blood samples (3 mice were pooled for a total of 24 in each group to obtain *n* = 8 per condition). Representative panels showing the purity of CD3^+^ and CD8^+^ cells. (N and O) Live-cell Ca^2+^ imaging showing representative images (after 300 s) of cells transduced with the mitochondrial Ca^2+^ indicator (GCaMP6f) and cytosolic (RCaMP), upon histamine stimulation, which was confirmed by quantitative analysis over time. (P and Q) OCR and ECAR measured using Seahorse XF in CON and TR CAR T cells with respective bar graphs showing the basal respiration (*n* = 6). (R1) Representative image of mtDNA, indicated by staining the cells anti-TFAM (Mitochondrial transcription factor A). (R2) Quantitative analysis of images (*n* = 11 images) was done and represented as integrated density of TFAM signal. (S) Fluorescent assessment of mitophagy by tracking LTDR/MTG ratios in CON and TR CAR T cells following FCCP treatment for 60 min. (T1) Representative images of CAR T cells, which were transduced with LC3-mCherry and mito-GFP showing co-localization of LC3 (red) with mitochondria (green). Blue arrowheads show co-localization, and white arrowheads indicate lack of co-localization. Cells were treated with FCCP for 30 min. (T2) Quantitative analysis of the LC3 images (*n* = 10 images). (U) p62, Beclin-1, and Atg14 expression measured by flow cytometry in CON and TR CAR T cells (*n* = 6). Data represents mean ± SEM. ∗*p* < 0.05; ∗∗*p* < 0.01; ∗∗∗*p* < 0.001; ∗∗∗∗*p* < 0.0001. A non-parametric t test and one-way ANOVA were used for statistical analysis. Scale bars: 10 μm.

CAR T cells typically exhibit limited persistence when exposed to recurring tumor burden, leading to metabolic shifts that contribute to functional decline.^29^ Therefore, our primary focus was to determine the mechanisms responsible for CAR T cell dysfunction upon tumor re-challenge (TR) *in vivo*. We set up a lymphoma tumor model using Raji cells and looked at markers associated with CAR T cell dysfunction (Figure 1A). CD19-targeting CAR T cells were generated using a 3^rd^-generation CAR plasmid and transduction of CD4/CD8^+^ T cells derived from the peripheral blood of healthy donors. These CAR T cells were then administered to mice on day 5, after the initial Raji administration. Flow cytometry was done, gating the human CD3^+^ (hCD3) cells derived from pooled peripheral blood collected at different time points (Figures 1B and S1). CAR T cell dysfunction was triggered upon TR, which was manifested by a reduction in Granzyme B and Perforin expression (Figures 1C, 1D, S2A, and S2B). These alterations were linked with an increase in markers of T cell exhaustion and a substantial decrease in the proliferation over time (Figures 1E–1G, S2C, and S2D). Further, we performed the co-culture of the isolated hCD3^+^ T cells with Raji cells *in vitro* and measured secretion of interferon (IFN)-γ and interleukin (IL)-2, which were significantly reduced in cells derived from TR conditions, demonstrating functional decline upon TR (Figures 1H and 1I). To identify T cell subsets linked to functional decline and exhaustion, we conducted immunophenotyping on CD4^+^ or CD8^+^ T cells and defined the T cell subsets as T naive (T_n_; CD45RO^−^ CCR7^+^), T stem cell memory (T_scm_; CD45RO^−^ CCR7^+^), T_cm_ (CD45RO^+^ CCR7^+^), T_em_ (CD45RO^+^ CCR7^−^), and T effector (T_eff_; CD45RO^−^ CCR7^−^). The analysis revealed a substantial decline in the generation of T_cm_ and T_n/scm_ cells accompanied by an increase in T_ex_ cells, specifically in the T_eff_ subset (Figures 1J, 1K, and S3). These results indicate that CAR T cells undergo functional decline over time upon TR, characterized by reduced T_m_ cell subsets, consequently impacting their long-term persistence.

Given the pivotal role of calcium signaling in T cell activation and function, we measured intracellular calcium [Ca^2+^]_i_ and mitochondrial [Ca2+]_mt_ levels in these cells. CD4^+^ and CD8^+^ CAR T cells were magnetically sorted from pooled blood samples of the tumor-bearing mice (day 28) (Figures 1L, 1M, S4A, and S4B). Interestingly, T cells obtained from TR mice showed no significant changes in [Ca^2+^]_i_; however, upon stimulation with histamine, an increase in the accumulation of [Ca^2+^]_mt_ was observed, which was more pronounced in CD8^+^ T cells as compared to CD4^+^ T cells (Figures 1N, 1O, S4C, and S4D). As Ca^2+^ accumulation is linked to mitochondrial permeability transition pore (mPTP) formation and cell death, we investigated the markers associated with these pathways and found an increase in the number of cells undergoing apoptosis, including upregulation of mitochondrial BAK and BAX and increase in mt-Ca^2+^ signal, which indicate mPTP formation (Figure S5). These findings suggest that CAR T cells obtained from TR animals are inherently more prone to cell death, potentially involving mitochondrial pathway.

Next, we conducted metabolic and functional analyses to assess mitochondria bioenergetic state. A pronounced decline in metabolic activity in cells from animals undergoing TR was observed, as indicated by a reduced oxygen consumption rate (OCR) and an increased extracellular acidification rate (ECAR), pointing toward a metabolic shift favoring glycolysis (Figures 1P, 1Q, S6A, and S6B). Additionally, a significant increase in mitochondrial mass, accompanied by a decrease in mitochondrial DNA (mtDNA), reduced mitochondrial membrane potential (ΔΨm), and elevated mitochondrial reactive oxygen species (mtROS), was observed, indicating compromised mitochondrial function (Figures 1R, S6C, and S7).

Since mitochondrial dysfunction along with accumulation is typically linked to impaired mitophagy,^30^ we performed mitophagy rate assay. This was done using FCCP in cells stained with MitoTracker Green (MTG) and LysoTracker Deep Red (LTDR) to determine the change in signal over time and presented as ratio of LTDR to MTG indicating mitophagy. The results showed that cells obtained from animals that had experienced TR exhibited an accumulation of dysfunctional mitochondria and underwent a decrease in the rate of mitophagy following FCCP treatment. (Figures 1S and S8A). To ascertain whether this phenomenon stemmed from mitochondrial dysfunction or a general autophagy defect, we also performed an autophagosome assay using plasmids encoding LC3-mCherry and mito-GFP, respectively, and assessed the extent of co-localization, as an indicator of autophagosome formation. The results showed a marked decrease in autophagosome formation, corroborated by reduced LC3 signal (Figures 1T and S8B), and a significant decrease in autophagy-related markers Atg14 and Beclin-1, key indicators of autophagy (Figures 1U and S8C–S8E). Notably, these changes were more pronounced in CD8^+^ T cells compared to CD4^+^ T cells, leading us to focus on CD8^+^ T cells in subsequent experiments. (Figures 1L–1U vs. S4–S8). Overall, these findings strongly indicate that CAR T cells undergo metabolic alterations during TR, resulting in the accumulation of dysfunctional mitochondria and a potential impairment in the autophagy process.

### Decline in mitophagy/autophagy induces defects in memory CAR T cell formation

**Figure 2:**
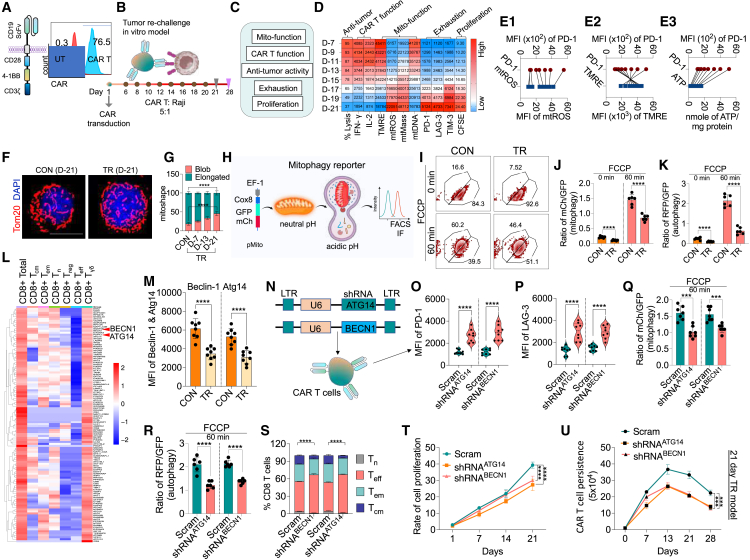
Decline in mitophagy/autophagy promotes dysfunction in CD8^+^ CAR T cells (A) Illustration of 3^rd^ generation CAR and flow cytometric analysis of CAR expression in CD8^+^ T cells. The y axis shows the total cell count, and the x axis represents the CAR-PE-positive cells. (B) CAR T cells were co-cultured with Raji cells (5:1 ratio) and then re-challenged with Raji cells every alternate day from day 6 to day 20 for functional analysis. (C) Analysis included CAR T cell multi-functionality, anti-tumor activity, exhaustion markers, and proliferation. (D) Heatmap of the various parameters showing anti-tumor activity by BLI and indicated as % lysis of tumor cells; expression of IFN- γ and IL-2 by ELISA; analysis of mtROS and TMRE by flow cytometry; mtDNA analysis by image quantitation; PD-1, LAG-3, and TIM-3 expression by flow cytometry; and cell proliferation by carboxyfluorescein succinimidyl ester (CFSE) staining (*n* = 6). The data are presented as MFI. (E1–E3) Correlation plots showing association between CAR T cell exhaustion and mitochondrial dysfunction. (F) Representative immunofluorescence images of Tom20-stained cells showing mitochondrial shape change in CAR T cells. (G) Quantification of various mitochondrial shapes in CON and TR groups (*n* = 6). (H) Schematic of reporter used to track mitophagy in live CAR T cells. Ratio of mCherry/GFP fluorescence indicates mitophagy progression. (I and J) Flow cytometric analysis showing mitophagy flux (mCherry/GFP ratio) over 60 min FCCP treatment. (K) Flow cytometry analysis showing the ratio of RFP to GFP as an indicator of autophagy (*n* = 6). (L) Transcriptomic analysis of existing patient-derived CAR T single-cell RNA sequencing data highlighting differential expression of autophagy-related genes, including BECN1 and ATG14, between CON and TR groups. (M) Beclin-1 and Atg14 expression in CAR T cells after TR suggesting alteration in autophagosome formation (*n* = 8). (N) Schematic of shRNA-mediated knockdown of ATG14 and BECN1 in CAR T cells. (O and P) MFI of PD-1 (O) and LAG-3 (P) measured by flow cytometry in CAR T cells transduced with scrambled (Scram) or shRNA targeting ATG14/BECN1 (*n* = 8). (Q and R) Ratio of mCherry/GFP or RFP/GFP fluorescence in mitophagy- and autophagy-deficient CAR T cells, respectively, following 60 min FCCP treatment (*n* = 7). (S) T cell subset analysis of CD8^+^ T cell (T_n_, T_cm_, T_em_, and T_eff_) in CAR T cells transduced with Scram or shRNA targeting ATG14/BECN1 (*n* = 6). (T) Proliferation rate over time in scrambled and shRNA knockdown groups (*n* = 6). (U) Analysis of CAR T cell persistence *in vitro*, comparing Scram and shRNA groups in the 21-day TR model (*n* = 6). Data represents mean ± SEM. ∗∗∗*p* < 0.001; ∗∗∗∗*p* < 0.0001. A non-parametric t test was used for statistical analysis. Scale bar: 10 μm.

To assess the impact of mitophagy/autophagy on CD8^+^ CAR T cells, we employed an *in vitro* model simulating *in vivo* TR. CD8^+^ T cells were isolated from peripheral blood of 5 healthy donors and used to generate 3^rd^ generation CAR T cells (Figure 2A). Subsequently, TR with Raji cells was performed with new cells added every 2 days (with media change) for 3 weeks, during which various parameters related to T cell function, exhaustion, and mitochondrial function were evaluated (Figures 2B and 2C). As anticipated, CAR T cells exhibited increase in exhaustion markers with TR over time, along with reduced CAR T cell functionality and anti-tumor activity (Figure 2D). Mitochondrial functional analysis revealed increased mtROS alongside a concurrent decrease in ΔΨm, indicating mitochondrial dysfunction. Moreover, consistent with *in vivo* observations, mitochondrial mass increased while a significant decline in mtDNA content occurred over time, correlating with reduced *in vitro* CAR T cell proliferation (Figure 2D), and a significant decline in ATP production was also observed over time (Figure S9A). Importantly, a close association between CAR T cell exhaustion, measured by PD-1, and a decline in mitochondrial functional parameters such as ΔΨm, mtROS, and ATP levels was observed after 3 weeks of TR (Figure 2E).

Previous studies established a link between mitochondrial shape change and T cell differentiation, with elongated mitochondria observed in T_m_ cells compared to punctate mitochondria in effector or exhausted cells.^17^ We evaluated mitochondrial shape changes over time and noted significant alterations in mitochondrial morphology, with a shift toward more punctate or blob-shaped mitochondria, contrasting with the more elongated shape observed in CON or during the early days of stimulation (Figures 2F and 2G). Given that the accumulation of fragmented mitochondria is a feature of impaired mitophagy, as observed in CAR T cells obtained from an *in vivo* model, we evaluated the rate of mitophagy in these cells using a mitophagy reporter assay based on the Cox8-EGFP-mCherry reporter^31^ (Figure 2H). TR resulted in a significant decline in mitophagy evidenced by a change in the GFP signal relative to mCherry, as measured by flow cytometry analysis at day 21 (Figures 2I and 2J). Given the crucial role of autophagophore formation in mitochondrial clearance observed *in vivo* (Figure 1), we investigated whether the observed defective mitophagy *in vitro* was associated with a broader reduction in autophagosome formation, using an autophagosome reporter similar to the one employed for mitophagy assessment (Figure S9B). Consistent with the findings from the mitophagy analysis, the autophagy reporter assay demonstrated impaired general autophagy in cells exposed to TR (Figures 2K and S9C). Notably, we also observed deficiencies in mitophagy and autophagy formation in CAR T cells stimulated by CD19-expressing NALM6 leukemia cells and the Daudi Burkitt’s lymphoma cell line, indicating that the defects were specific to the stimulation rather than the type of cancer cells (Figures S9D–S9G).

To evaluate the autophagy gene expression status across different subsets of CAR T cells, we analyzed existing single-cell RNA sequencing (scRNA-seq) data from patients who had received CD19 CAR T cell therapy.^32^ We observed a notable expression of autophagy genes in memory subsets of both CD8^+^ and CD4^+^ T cells compared to T_eff_ and regulatory T (T_reg_) cells (Figures 2L and S10). Based on their expression profiles, we identified ATG14 and BECN1 as significantly downregulated genes in T_eff_ and T_reg_ cells, while their levels were maintained in T_m_ subsets. We functionally validated these markers in a TR model, at day 21 after TR, and found a substantial decrease in both Atg14 and Beclin-1 protein expression (Figures 2M, S11A, and S11B), suggesting that a general alteration in autophagosome formation may contribute to the accumulation of dysfunctional mitochondria in T_m_ cells. To assess the impact of autophagy on CAR T cells, we employed an short hairpin RNA (shRNA)-based strategy to suppress the expression of these two proteins after day 7 of transduction, following a single tumor challenge on day 5 (Figures 2N, S11C, and S11D). Knockdown (KD) of ATG14 and BECN1 recapitulated TR conditions, leading to the expression of PD-1 and LAG-3 with a simultaneous decrease in IL-2 secretion and Granzyme B expression (Figures 2O, 2P, S11E, and S11F). Moreover, the KD of these proteins was linked to reduced mitophagy and autophagosome formation (Figures 2Q and 2R), accompanied by a significant decrease in the formation and proliferation of CAR T memory cells (Figures 2S and 2T). Notably, in a repeated TR experiment, the KD further exacerbated the decline in CAR T cell persistence (Figure 2U). These findings strengthened the evidence from animal models that impaired autophagy led to the accumulation of damaged mitochondria, resulting in CAR T cell exhaustion and subsequently contributing to a reduction in T_m_ cell formation and long-term persistence.

### SG and UrA synergistically reduce CAR T cell dysfunction and promote long-term persistence

**Figure 3:**
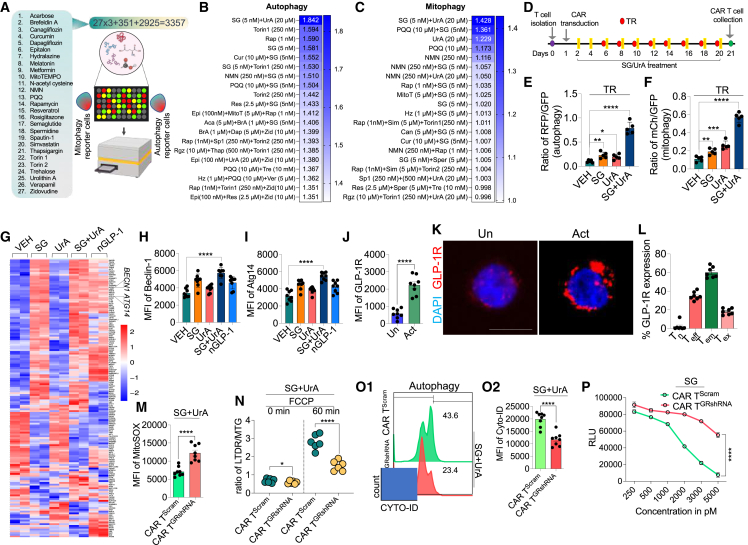
SG and UrA enhance autophagy and reduce CAR T cell dysfunction (A) Schematics of workflow of test compound screening for autophagy and mitophagy using reporter Jurkat stable cells. (B and C) Heatmaps showing the effects of single, combinations, and dose titrations on autophagy (B) and mitophagy (C), assessed using GFP-RFP reporter systems. The blue scale indicates the intensity of activity (*n* = 3, mean). (D) Schematic of T cell isolation, CAR T cell transduction, drug treatment (SG and UrA), and TR (every alternate day from day 6 to day 20). CAR T cell collection was done on day 21. (E and F) Flow cytometric analysis of RFP/GFP ratios for autophagy (E) and mCherry/GFP for mitophagy (F) in TR CAR T cells treated with VEH, SG, UrA, or SG + UrA (*n* = 5). (G) Transcriptomic analysis of autophagy-related gene expression in TR CAR T cells, including Beclin-1 and Atg14, across different treatment conditions (SG, UrA, SG + UrA, and nGLP-1). (H and I) MFI of Beclin-1(H) and Atg14 (I) in CAR T cells treated with VEH, SG, UrA, SG + UrA, or nGLP-1 (H: *n* = 7; I: *n* = 8). (J) GLP-1R expression in activated (Act) vs. unactivated (Un) CAR T cells measured by flow cytometry (*n* = 8). (K) Representative images showing GLP-1R (red) and DAPI (blue) staining in activated and unactivated CAR T cells. (L) Flow cytometric analysis of GLP-1R expression across different T cell subsets (T_n_, T_eff_, T_cm_, and T_ex_) in activated CAR T cells (*n* = 7). (M) MFI of MitoSOX staining in CAR T cells transduced with scrambled shRNA or GRshRNA and treated with SG + UrA (*n* = 8). (N) Measurement of mitophagy flux in CAR T cells transduced with scrambled control (Scram) or GRshRNA using LTDR/MTG ratios after FCCP treatment (*n* = 6). (O1) Similarly, flow cytometric analysis of CYTO-ID showing autophagy. (O2) MFI of CYTO-ID (*n* = 8). (P) The cAMP activity assay with relative light unit (RLU) depicted as the inverse relationship between cAMP concentration and RLU (*n* = 6), with varying concentrations of SG. Data represent mean ± SE; from three independent experiments. ∗*p* < 0.01; ∗∗*p* < 0.01; ∗∗∗*p* < 0.001; ∗∗∗∗*p* < 0.0001. A non-parametric t test and two-way ANOVA were used for statistical analysis between groups. Scale bar: 10 μm.

We screened 27 validated compounds related to autophagy, mitophagy, and mitochondrial metabolism to address CAR T cell mitophagy/autophagy deficits. The screening included SG, a weight-loss drug with potential autophagy effects. Using autophagy and mitophagy reporter Jurkat T cell lines, we tested the compounds individually at three doses, in pairs, and in triple combinations, totaling 3,357 combinations (Figure 3A and Table S1). The initial screening identified combinations of SG, UrA, and other autophagy and mitophagy inducers that significantly activated both general autophagy and selective mitophagy (Figures 3B and 3C). The top five compounds from the screening, which influenced both mitophagy and autophagy induction, were then evaluated in CAR T cells from healthy donors using flow cytometry. The analysis showed increased autophagy and mitophagy, with the highest levels observed in cells treated with the SG and UrA combination, compared to other drug combinations (Figures S12A and S12B). We then tested various dose combinations of SG and UrA and found that the combination of SG (5 nM) and UrA (10 μM) was the most effective, with no impact on cell death. Individually, these drugs were less effective in inducing autophagy or mitophagy (Figures S12C–S12E). Importantly, this drug combination had no effect on CD4^+^ T cell polarization (Figure S13). Therefore, it was selected for further studies.

We evaluated the effects of SG + UrA in the TR model, initially focusing on autophagy and mitophagy induction (Figure 3D). CAR T cells were treated alternately with the drugs from day 2 after CAR transduction until day 21. When TR CAR T cells were cultured *in vitro* for 21 days with SG, UrA, or their combination, SG + UrA significantly increased mitophagy and autophagy compared to individual treatments, suggesting that it effectively alleviates deficiencies in these pathways (Figures 3E, 3F, S12A, and S12B). By day 21, mitochondrial function was restored, along with the CAR T cell functional phenotype (Figures S14C–S14F). Notably, cells cultured with SG + UrA showed reduced exhaustion markers and an increased number of T_m_ cells (Figures S14G–S14I). In the TR model, a persistence assay conducted up to day 28 (TR until day 20) revealed that SG + UrA significantly enhanced CAR T cell persistence (Figure S14J). To evaluate anti-tumor efficacy, a co-culture assay with Raji cells was performed. The combination therapy significantly enhanced anti-tumor activity compared to individual drug treatments (Figure S15A). This augmented anti-tumor response, accompanied by increased proliferation, was further corroborated through live-cell imaging of the co-culture (Videos S1 and S2). The potentiated anti-tumor effects of drug-treated CAR T cells were validated in a leukemia model utilizing NALM6 cells, a multiple myeloma model with BCMA-directed CAR T cells, and a solid tumor model employing GD2-directed CAR T cells against SH-SY5Y neuroblastoma cells (Figures S15B–S15D). However, none of these molecules, either alone or in combination, had a significant effect on cell death in these tumor cell lines at the chosen concentrations (Figures S15E–S15H). Importantly, TR assay was conducted in the CAR T cells generated from the blood of 3 leukemia patients against the B cells obtained from the same patients. The TR CAR T cells were able to maintain a certain level of anti-tumor activity and persistence over 28 days, which was robustly enhanced in presence of SG + UrA (Figure S16). Overall, these results demonstrate that the SG and UrA combination effectively recalibrates CAR T cell metabolic functions, primarily by inducing autophagy and mitophagy.

Video S1. Live-cell imaging of CAR T cells co-cultured with Raji cells (mCherry, red) in regular media for 12 h, related to Figure S15A

Video S2. Live-cell imaging of CAR T cells co-cultured with Raji cells (mCherry, red) after 7-day culture in SG + UrA after CAR transduction, for 12 h, related to Figure S15A

### GLP-1 induces autophagy through mTOR activation, whereas UrA activates Atg4b

Based on this finding, we next investigated the molecular mechanisms underlying the action of these drug combinations. We conducted RNA sequencing (RNA-seq) in 21-day TR CAR T cells cultured with these drug combinations, along with naturally occurring GLP-1RA (nGLP-1 hereafter), which is endogenously secreted by intestinal L-cells. The RNA-seq results revealed a significant reduction in autophagy-related gene expression in CAR T cells, a trend that was reversed upon treatment with SG (SG [5 nM]) or nGLP-1 (SG [5 nM]). UrA alone did not enhance autophagy gene expression as robustly as the SG + UrA combination (Figure 3G). Protein-level validation of key genes showed increased expression of Atg14 and Beclin-1 following SG or nGLP-1 treatment (Figures 3H, 3I, S17A, and S17B) but not with UrA.

To elucidate the potential mechanism, we investigated the expression of their respective receptor (GLP-1R) across different T cell subsets. While GLP-1R expression was low in resting T cells, stimulation via CD3/CD28 resulted in an increase in its expression (Figures 3J and 3K). Additionally, we examined GLP-1R expression patterns after TR, observing predominant expression in T_m_ cells and lower levels in T_ex_ or T_eff_ cells (Figure 3L). To confirm the impact of GLP-1 on autophagy, GLP-1R KD was performed using lentiviral-mediated shRNA delivery (Figure S17). KD reduced effects of SG on mitochondrial function (Figure 3M) and impaired mitophagy and autophagy clearance in the TR model at day 21 (Figures 3N and 3O). Additionally, SG-induced cyclic AMP (cAMP) levels were diminished in KD cells (Figure 3P). These findings suggest that GLP-1R activation mediates autophagy induction in CAR T cells, with the intracellular cAMP pathway playing a crucial role in this signaling process.

**Figure 4:**
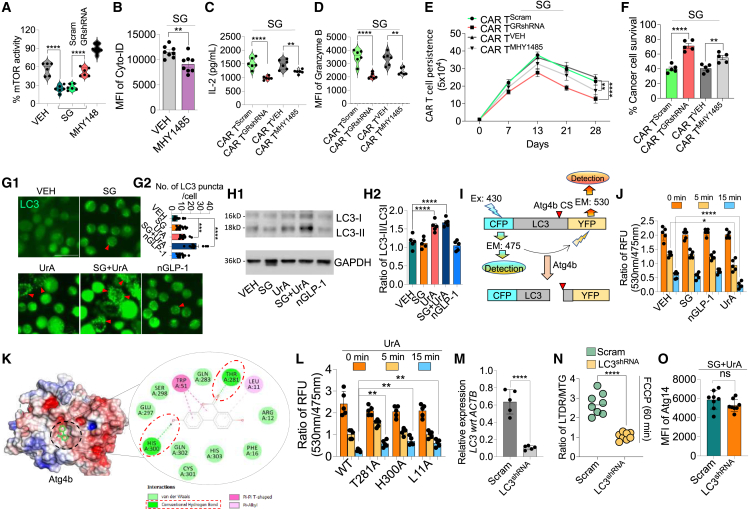
Analysis of mTOR activity, autophagy, and CAR T cell function (A) mTOR activity measured by flow cytometry in CAR T cells treated with VEH, SG (Scram and GRshRNA transduced), and MHY1485 as positive control (*n* = 6). (B) MFI of CYTO-ID (*n* = 6). (C and D) IL-2 secretion (C) and Granzyme B expression (D) measured by ELISA and flow cytometry, respectively, in CAR T cells transduced with Scram and GRshRNA or treated with VEH or MHY1485 cultured in the presence of SG (*n* = 6). (E) Analysis of CAR T cell persistence under various conditions. (F) Similarly, percentage of cancer cell survival after co-culture with CAR T cells (*n* = 5). (G1 and G2) Representative images of LC3 staining (green) showing autophagosome formation in CAR T cells treated with VEH, SG, UrA, SG + UrA, and nGLP-1. Red arrows indicate LC3 puncta. Quantification of LC3 puncta per cell is shown in (G2) (*n* = 14 images). (H1 and H2) Western blot analysis of LC3-I and LC3-II expression in CAR T cells. Quantification of LC3-II/LC3-I ratio is shown in (H2) (*n* = 5). (I) Schematic representation of the FRET-LC3II plasmid construction. The relative fluorescence unit (RFU) ratio of 530–475 nm (YFP/CFP) was calculated to quantify the extent of LC3B cleavage, where a decrease in the ratio indicated increased substrate cleavage. (J) FRET analysis using a CFP-LC3-YFP reporter. *In vitro* assay conducted by incubating purified Atg4b protein with CFP-LC3-YFP in the presence of VEH, UrA, SG, or nGLP-1. FRET signals were recorded at 0, 5, and 15 min (*n* = 5). (K) Structural model of Atg4b interaction with its substrates, highlighting key amino acids involved in the interaction, based on *in silico* docking predictions. (L) Fluorescence intensity (RFU ratio 530 nm/475 nm) over time in CAR T cells expressing wild-type and mutated forms (T28A, H300A, and L11A) of Atg4b, treated with UrA (*n* = 5). (M) Relative expression of LC3 normalized to ACTB in CAR T cells transduced with Scram or LC3-targeted shRNA (*n* = 5). (N) Ratio of LTDR/MTG after FCCP treatment for 60 min (*n* = 8). (O) MFI of Atg14 in CAR T cells after treatment (*n* = 6). Data represent mean ± SEM.∗*p* < 0.01; ∗∗*p* < 0.01; ∗∗∗∗*p* < 0.0001; (ns, not significant). A non-parametric t test was used for statistical analysis. Scale bar: 50 μm.

To determine whether GLP-1/GLP-1R signaling influences mTOR activity, a key regulator of autophagy inhibition, we performed an assay using immunoprecipitated mTOR from T cells. SG induction reduced mTOR activity, an effect that remained unchanged in GLP-1R KD cells, suggesting that GLP-1-mediated autophagy induction involves elevated cAMP levels leading to mTOR inhibition (Figure 4A). This was further supported by the observation that MHY1485, a known mTOR activator, counteracted SG’s effect on autophagy induction (Figure 4B). GLP-1R KD cells and those treated with MHY1485 showed a significant reduction in CAR T cell function, though the decline was less pronounced in the latter condition. This was evidenced by decreased IL-2 secretion and Granzyme B expression (Figures 4C and 4D). The functional decline correlated with reduced *in vivo* persistence and a notable decrease in *in vitro* anti-tumor activity (Figures 4E and 4F). Cells treated with SG and transduced with shRNA for mTOR had a baseline increase in autophagy, which was not significantly increased upon mTOR KD. This suggests that GLP-1R activation mediates its effects through the mTOR pathway (Figure S18). Overall, these results indicate that GLP-1R activation induces autophagy primarily through mTOR inhibition. However, since mTOR activation by MHY1485 did not fully eliminate the effects of GLP-1R activation, additional mechanisms may contribute to autophagy induction and the maintenance of the memory stem cell phenotype in CAR T cells. One such mechanism could be the transcriptional activation of key genes like ATG14 and BECN1, as shown in Figure 3G.

Conversely, CAR T cells transduced with LC3-GFP and treated with UrA exhibited LC3II maturation, indicated by puncta formation and increased expression. This effect remained unchanged in the presence of SG or nGLP-1 (Figures 4G and 4H). To find whether UrA-mediated effect on LC3 is via induction of Atg4b (key protein involved in LC3 maturation), we conducted fluorescence resonance energy transfer (FRET) analysis using a CFP-LC3-YFP reporter, as described previously^33^ (Figure 4I). An *in vitro* cuvette assay was performed with the purified individual protein of Atg4b incubated with a CFP-LC3-YFP reporter in the presence of UrA, SG, or nGLP-1, and the FRET signals were detected over 15 min. The results showed that UrA but not SG or GLP-1RA specifically and significantly activated Atg4b-mediated cleavage of LC3 (Figure 4J). Molecular docking confirmed the binding of UrA with Atg4b (Figure 4K). Interestingly, the effect of UrA was slightly diminished in the Atg4b mutants, which exhibited lower binding for UrA (Figures 4L and S19). KD of LC3 reduced mitophagy upon SG + UrA treatment but had no effect on Atg14 levels (Figures 4M–4O). These results suggest that UrA induces LC3 maturation, potentially involving Atg4b. Overall, SG broadly enhances autophagy by inhibiting mTOR and activating transcription, while UrA specifically promotes LC3 maturation, which is essential for mitophagy. This aligns with previous studies on UrA’s function.^34^ These findings highlight the synergistic role of SG and UrA in eliminating dysfunctional mitochondria. Notably, nGLP-1 exhibited a similar effect to SG, though to a lesser extent, possibly due to SG’s more stable structure.^35^

### MCAR T-1 cells enhance anti-tumor activity and long-term persistence of CAR T cells

**Figure 5:**
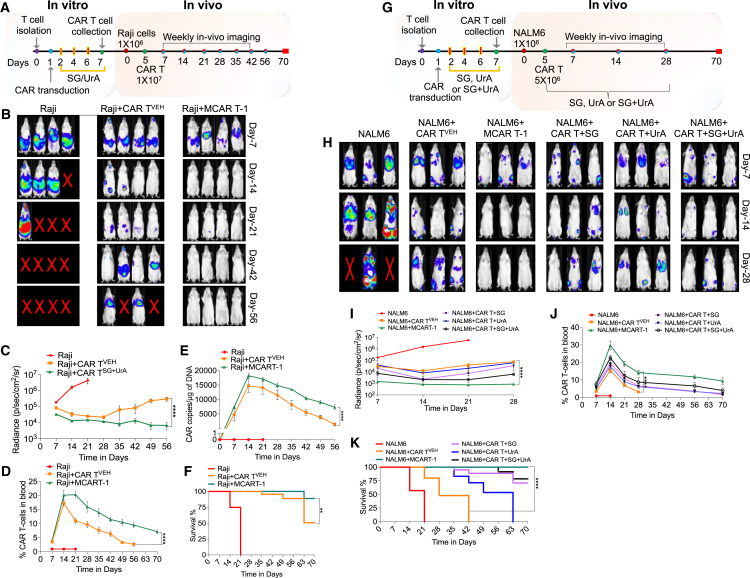
Anti-tumor activity and CAR T cell persistence after treatment with SG, UrA, or their combination in Raji and NALM6 tumor models (A) Schematic of the Raji tumor model. CAR T cells were isolated, transduced, and treated with SG + UrA *in vitro* (MCAR T-1), maintained for 7 days. Raji cells were injected into mice, followed by CAR T cell infusion (1 × 10^7^ cells) on day 5. Weekly *in vivo* imaging was conducted from day 7 to day 56, and weekly blood collection till day 70. (B) Representative images of tumor burden in mice treated over time (days 7, 14, 21, 42, and 56) (*n* = 4). (C) Quantification of bioluminescence radiance (photons/sec/cm^2^/sr) in Raji tumor-bearing mice over time (*n* = 5; at day 21; *n* = 1 for Raji group). (D) CAR T cell persistence in the blood at various time points till day 70 (*n* = 5). (E) Quantification of CAR T cell DNA copies per μg in blood samples over time. (F) Kaplan-Meier survival curve showing the survival of mice (*n* = 5). (G) Schematic of the NALM6 tumor model, with similar isolation, transduction, and treatment of CAR T cells (VEH, SG, UrA, and SG + UrA [MCAR T-1]) or direct administration of SG, UrA, or SG + UrA to mice. NALM6 cells were injected into mice, followed by CAR T cell infusion (5 × 10^6^ cells) on day 5. *In vivo* imaging was performed weekly from day 7 to day 56. (H) Representative images of tumor burden in mice over time (days 7, 14, and 28; *n* = 3 at day 21; *n* = 1 for Raji group). (I) Quantification of bioluminescence radiance (photons/sec/cm^2^/sr) in NALM6 tumor-bearing mice (*n* = 5). (J) Percentage of CAR T cells in blood at different time points (*n* = 5). (K) Survival plot of the mice over 70-day time period (*n* = 5). Data represent mean ± SEM. ∗∗∗∗*p* < 0.0001. A non-parametric t test and the Mantel-Cox test were used for statistical analysis.

To evaluate the impact of SG + UrA on metabolically altered CAR T cells, we assessed their anti-tumor effect using a lymphoma mouse model (Figure 5A). CAR T cells targeting CD19 were cultured with SG + UrA for 7 days before being administered to mice and compared to cells cultured under normal media conditions. Mice were given vehicle-treated CAR T cells (CAR T^VEH^) or CAR T cells that were treated with SG + UrA (referred as metabolically enhanced CAR T; MCAR T-1) on day 5 after Raji tumor injection. We observed an enhancement in anti-tumor activity in mice treated with MCAR T-1, evidenced by a significant reduction in bioluminescence imaging (BLI) compared to those receiving CAR T^VEH^ alone (Figure 5B). Quantitative analysis of tumor signals showed a significant reduction in tumor size over time in the MCAR T-1 treatment group. In contrast, mice treated with CAR T^VEH^ cells experienced relapse, with a statistically significant difference observed (Figure 5C). We investigated whether the sustained anti-tumor effect in the treatment group was due to prolonged CAR T cell presence. CAR T cells were measured in pooled blood samples, and CAR copy number was quantified. The MCAR T-1-treated group showed a higher percentage of CAR T cells and an increased CAR copy number (Figures 5D and 5E). We also found that mice administered MCAR T-1 exhibited prolonged survival compared to those receiving CAR T^VEH^ (Figure 5F). Overall, these results indicate that SG + UrA significantly enhances long-term persistence and thereby improves the anti-tumor activity of CAR T cells.

To assess whether the long-term persistence and anti-tumor activity of MCAR T-1 cells were effective at lower doses and in other cancer models and to understand the impact of direct administration of these molecules, we employed the NALM6 leukemia model. SG and UrA were tested individually and in combination by directly administering these molecules to the mice (Figure 5G). As observed with Raji cells, MCAR T-1 cells maintained anti-tumor activity even at lower doses compared to those used with Raji cells (Figures 5H and 5I) and exhibited prolonged persistence (Figure 5J). Direct administration of SG or UrA alone had a limited impact. However, their combination provided a slight improvement in anti-tumor activity and CAR T cell persistence. Still, this effect—whether from SG, UrA, or their combination—was less pronounced than that observed with MCAR T-1 cells (CAR T cells cultured *in vitro* with these molecules) (Figures 5G–5I). The efficacy of MCAR T-1 cells at a low dose was tested in the Raji model, demonstrating that, even at low doses, these cells exhibited significantly better efficacy than CAR T cells alone (Figure S20). These findings suggest that SG + UrA exerts a lasting and synergistic effect on CAR T cell persistence, likely by promoting the generation of T_m_ cells through autophagy induction and the removal of dysfunctional mitochondria during T cell differentiation.

### CAR plasmids encoding natural GLP-1 peptide induce long-term persistence and prevent T cell dysfunction

**Figure 6:**
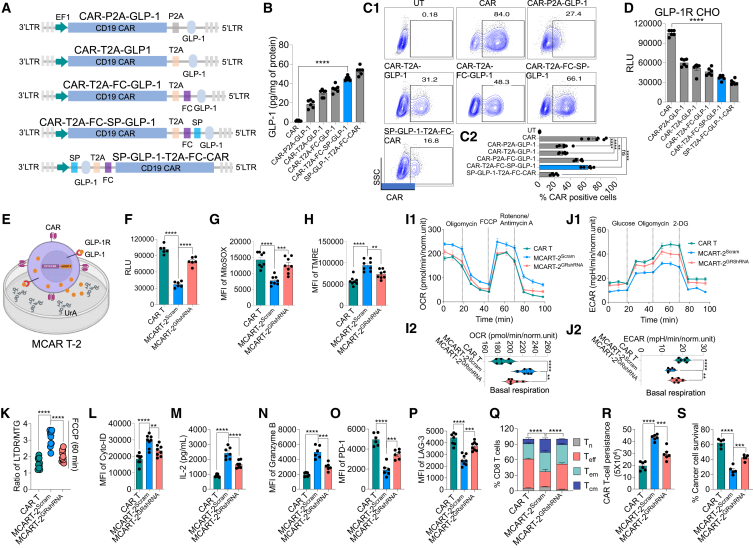
Engineering and evaluation of nGLP-1-secreting UrA-treated CAR T cells for autophagy, mitophagy, and anti-tumor activity (A) Various GLP-1 CAR constructs with nGLP-1 positioned at the N or C terminus of the CAR molecule, separated by P2A or T2A cleavage sites, including versions with a furin cleavage (FC) site and secretory signal peptide (SP). (B) Levels of GLP-1 secretion from CAR T cells with different constructs measured by ELISA at day 7 after transduction. (*n* = 6). (C1 and C2) Contour plots and analysis showing CAR expression and percentage positive CAR-expressing cells (*n* = 6). (D) cAMP levels in GLP-1R-expressing CHO cells exposed to supernatants (collected after 72 h of transduction) from CAR T cells transduced with various GLP-1 CAR constructs (*n* = 6). (E) Schematic of scGLP-1/CAR T cell treatment with UrA (referred as MCAR T-2). (F) Measurement of cAMP release in CAR T cells or scGLP-1/CAR T cells treated with the latter also transduced with Scram or GRshRNA (*n* = 6). (G and H) Flow cytometric analysis showing MFI of mtROS (G) and ΔΨm (TMRE, H) in CAR T or MCAR T-2 cells transduced with Scram or GRshRNA (*n* = 8). (I) OCR measured by Seahorse XF analyzer; the bar graph shows basal respiration (*n* = 6). (J) Similarly, ECAR measured by Seahorse XF analyzer; the bar graph shows basal respiration (*n* = 6). (K and L) Ratio of LTDR/MTG (K) and percentage of autophagy (L). (M and N) IL-2 secretion (M) and Granzyme B expression (N) measured by ELISA and flow cytometry (*n* = 6). (O–Q) Expression of PD-1 (O) and LAG-3 (P) measured by flow cytometry and represented as MFI. T cell subset analysis (Q) shows the percentage of different memory subsets (T_n_, T_scm_, T_cm_, and T_em_) after treatment (*n* = 6). (R and S) Persistence of CAR T cells (R) and percentage cancer cell survival (S) in co-culture assays (*n* = 5 or 6). Data represent mean ± SEM. ∗∗*p* < 0.01; ∗∗∗*p* < 0.001; ∗∗∗∗*p* < 0.0001; ns, not significant. A non-parametric t test, one-way ANOVA, and the Mantel-Cox test were used for statistical analysis.

To develop a clinically viable approach, we engineered CARs that secrete GLP-1. Initially, we compared the activation levels induced by nGLP-1 and SG in a stable GLP-1R-expressing Chinese hamster ovary (CHO) cell line, noting that SG robustly activated cAMP, while nGLP-1 showed a similar effect, albeit to a lesser extent (Figure S21A). We proceeded with nGLP-1 for CAR design, as SG contains non-natural amino acids. Various constructs were created with nGLP-1 positioned either at the N terminus or C terminus of the third-generation CAR molecule, separated by a T2A or P2A peptide cleavage site. Plasmid designs were further optimized by incorporating a furin cleavage site and a secretory signal peptide (Figure 6A). Initial screening measured secreted nGLP-1 in CAR T cell supernatants at day 7 after transduction, revealing that placing nGLP-1 immediately after the promoter significantly increased its secretion (Figure 6B), albeit with reduced CAR expression. Conversely, placing nGLP-1 after the T2A and furin cleavage site with its own signal peptide resulted in robust CAR expression and significant nGLP-1 release (Figure 6C). To confirm the functionality of secreted nGLP-1, we conducted *in vitro* assay using GLP-1R-expressing CHO cells. The assay demonstrated increased cAMP levels when exposed to media containing nGLP-1 obtained from GLP-1-CAR-transduced T cells (Figure 6D). Thus, we selected the construct with nGLP-1 after the T2A and furin cleavage site, along with its own secretory signal, for further evaluation, referred to as scGLP-1-CAR.

Following that, we conducted *in vitro* assessments of scGLP-1-CAR. Guided by the autophagy/mitophagy screening outcomes shown in Figure 3, we supplemented UrA in the culture medium and used GLP-1R shRNA to focus specifically on the impact of nGLP-1 in the TR model after day 21 (Figure 6E). Notably, scGLP-1/CAR T cells cultured in UrA-containing media (referred to as MCAR T-2; metabolically optimized CAR T cells) exhibited increased cAMP release (Figure 6F). The synergistic effect of nGLP-1 and UrA significantly improved mitochondrial function and promoted mitochondrial-based metabolism (Figures 6G–6I). Notably, both mitophagy and autophagy were enhanced in MCAR T-2, whereas their activation was markedly reduced in GLP-1R KD cells, highlighting the essential role of these pathways in maintaining mitochondrial health and metabolic activity (Figures 6J and 6K). Subsequently, we evaluated the functional outcomes and observed increased IL-2 and Granzyme B expression in MCAR T-2 cells, which was attenuated by GLP-1R KD (Figures 6L and 6M). We noted a significant reduction in exhaustion markers and an increase in stem cell markers (Figures 6N–6P). These observations were further corroborated by the long-term persistence and robust anti-tumor activity against Raji cells (Figures 6Q and 6R). However, these effects were diminished upon GLP-1R KD (Figures 6L–6R), similar to what observed *in vitro* (Figure 4). To determine whether the anti-tumor activity was due to the enhanced activity of the MCAR T-2 cells and not due to scGLP-1 and UrA directly targeting Raji cells, we obtained the supernatant from MCAR T-2 cells and incubated with Raji cells. No significant cell death was observed, suggesting that the effect of these molecules is mediated via directly enhancing the signaling/function in CAR T cells (Figures S21B–S21D). These findings demonstrate that MCAR T-2 cells exhibit enhanced anti-tumor activity and prolonged persistence by activating autophagy/mitophagy pathways through GLP-1R receptor activation, with these effects being synergistically amplified by UrA.

### MCAR T-2 cells induce robust anti-tumor activity and long-term persistence *in vivo*

**Figure 7:**
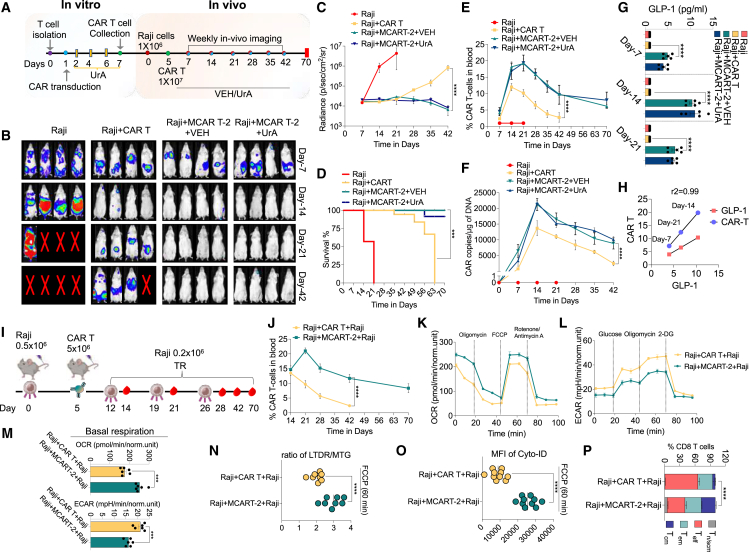
MCAR T-2 cells induce robust anti-tumor activity and improve long-term persistence *in vivo* (A) Schematic of the *in vitro* and *in vivo* experimental setup for Raji tumor-bearing mice. CAR T cells were transduced and cultured in UrA-containing media (MCAR T-2). Raji cells were injected into mice on day 0, followed by CAR T cell infusion on day 5. (B) Representative BLI images of mice showing tumor burden over time. Images were taken at days 7, 14, 21, and 42 (*n* = 4). (C) Quantification of tumor burden (radiance) over time (photons/sec/cm^2^/sr). (D) Kaplan-Meier survival curve. (E) Percentage of CAR T cells in blood over time. (F) CAR DNA copies measured by qPCR at different time points (*n* = 5 at day 21; *n* = 1 for Raji group). (G) GLP-1 levels in serum measured at days 7, 14, and 21 by ELISA. (H) Correlation of GLP-1 levels with CAR T cell percentage in blood at different time points (r^2^ = 0.99) (*n* = 5). (I) Schematic of the tumor re-challenge model; mice were injected with Raji cells and treated with CAR T or MCAR T-2 cells on day 5. TR was induced on days 12, 19, and 26. (J) Percentage of CAR T cells in blood after TR. (K and L) (K) OCR and (L) ECAR measured by Seahorse XF analyzer, showing improved mitochondrial function in MCAR T-2-treated mice (*n* = 5). (M) Basal respiration plots of OCR and ECAR (*n* = 6). (N and O) Mitophagy and autophagy analysis. (N) Ratio of LTDR/MTG in CAR T or scGLP-1/CAR T^UrA^-treated mice. (O) Percentage of autophagy-positive cells (*n* = 8). (P) Flow cytometric analysis of T cell subsets (Tn, Tscm, Tcm, and Tem) in CAR T or scGLP-1/CAR T^UrA^-treated mice after TR (*n* = 5). Data represent mean ± SEM. ∗∗∗*p* < 0.001; ∗∗∗∗*p* < 0.0001. A non-parametric t test, one-way ANOVA, and the Mantel-Cox test were used for statistical analysis.

To evaluate the *in vivo* impact of MCAR T-2 cells, we conducted experiments using the Raji tumor model. MCAR T-2 cells were generated by culturing scGLP-1/CAR T cells in UrA-containing media, as described earlier. For comparative analysis, one group of mice received daily UrA feeding following MCAR T-2 cell administration. Consistent with our *in vitro* findings, there was enhancement in tumor killing efficacy when MCAR T-2 cells were utilized (Figures 7B and 7C). Importantly, MCAR T-2 cells demonstrated significantly enhanced overall survival of the mice (Figure 7D). However, direct administration of UrA to the animals did not confer any additional advantage in terms of anti-tumor activity or survival. The increased survival of the mice was correlated with detectable levels of CAR T cells and CAR DNA in the blood (Figures 7E and 7F). Further, scGLP-1 was detectable in the blood serum of the mice, showing correlation with the presence of CAR T cells (Figures 7G and 7H). Evaluation of tissue toxicity revealed that MCAR T-2 cells did not induce any adverse effects (Figure S23). Importantly, to determine the effect of MCAR T-2 on cytokine release syndrome (CRS), which is a major concern with CAR T cell therapy, we generated a CRS model, based on previous studies.^36^ We observed a significant decrease in the major cytokines related to CRS such as murine IL-6; IL-1β; CCL2, and tumor necrosis factor alpha (TNF-α) compared to vehicle-treated CAR T cells (Figure S22).

As CAR T cells exhibit dysfunction after repeated TR, we examined the effects of MCAR T-2 cells and their response during TR *in vivo* (Figure 7I). Notably, MCAR T-2 cells exhibited increased *in vivo* persistence compared to CAR T cells alone (Figure 7J). Evaluation of mitochondrial parameters revealed improved mitochondrial functionality along with increased autophagy/mitophagy induction (Figures 7K–7N). These changes correlated with an expanded T_m_ cell population (Figure 7O). These findings highlight the enhanced safety, anti-tumor efficacy, and prolonged persistence of GLP-1 CAR T cells compared to conventional CAR T cells. Notably, incorporating UrA during the *in vitro* manufacturing of scGLP-1/CAR T cells helps preserve mitochondrial integrity, thereby reducing CAR T cell exhaustion.

## Discussion

Compromised metabolism and mitochondrial dysfunction are key factors limiting the effectiveness of T cell immunotherapy.^9^^,^^10^^,^^37^^,^^38^ CAR T cell therapy, which involves genetically modifying T cells to express CARs targeting specific tumor antigens, has revolutionized cancer treatment.^1^^,^^38^ However, functional decline and limited persistence, particularly under repeated tumor exposure/TR, remain significant hurdles.^7^^,^^39^ In this study, we developed metabolically reprogrammed CAR T (MCAR T) cells that resist TR and exhibit durable persistence. We showed that CAR T cell dysfunction during TR is primarily caused by impaired mitophagy and reduced autophagy, leading to limited persistence.

Mitochondrial metabolism is critical for T_eff_ cell differentiation, memory and stem-like T cell maintenance, and exhaustion prevention.^8^^,^^9^^,^^10^^,^^11^^,^^12^ Persistent antigen exposure imposes significant metabolic demands on T_eff_ cells, often pushing them toward glycolysis, which drives exhaustion rather than differentiation into long-lasting T_m_ cells. Additionally, deficiencies in the autophagy pathway diminish T cell function and impair T_m_ cell formation.^24^^,^^25^^,^^40^ The coordination of autophagic signaling with mitochondrial metabolism is thus essential for the development and persistence of long-lasting T_m_ cells, as validated in our *in vitro* and *in vivo* TR models of CAR T cells.

To identify therapeutic targets that enhance autophagy and mitophagy, we screened compounds affecting metabolism, autophagy, and mitophagy. Among these, SG (a GLP-1 receptor agonist) and UrA (promotes mitophagy) were particularly effective.^34^^,^^41^^,^^42^ CAR T cells cultured with SG and UrA (MCAR T-1) showed synergistic increases in autophagy and mitophagy, evidenced by enhanced autophagosome formation and clearance of dysfunctional mitochondria. This rejuvenation improved mitochondrial function, reduced exhaustion, and enhanced CAR T cell persistence and functionality. MCAR T-1 cells exhibited robust anti-tumor activity by reversing TR-mediated metabolic changes, as demonstrated in lymphoma and leukemia models. Importantly, metabolic reprogramming with SG and UrA significantly enhanced CAR T cell function and reversed tumor-fighting limitations. These findings align with previous studies on metabolic interventions improving T cell function.^12^^,^^43^

Enhancing mitophagy is emerging as a critical factor in rejuvenating T cell metabolism and functionality.^18^^,^^44^^,^^45^ Furthermore, these metabolic improvements may reduce the required CAR T cell dose while maintaining therapeutic efficacy due to enhanced persistence and T_m_ cell generation. We observed that halving the MCAR T-1 dose in both leukemia and lymphoma tumor models still achieved similar anti-tumor activity, and the mice survived beyond 70 days, compared to 40 days for the conventional CAR T cell group. Thus, the effect of SG and UrA on autophagy and mitophagy enhances CAR T cell functionality, potentially lowering toxicities like CRS.^46^

When used alone *in vitro* or *in vivo*, SG or its natural ligand nGLP-1 (collectively GLP-1RA) or UrA showed limited efficacy, with no direct tumor cell killing at the tested concentrations. This suggests that their effects are mediated through CAR T cell metabolism. Supporting this, GLP-1R KD significantly reduced the metabolic benefits of these molecules. Building on these findings, we designed a CAR molecule secreting nGLP-1 (scGLP-1 CAR T or MCAR T-2 cells), eliminating the need for exogenous GLP-1RA. These MCAR T-2 cells enhanced autophagy, mitophagy, and memory cell generation while boosting anti-tumor activity in UrA-containing media, comparable to MCAR T-1. Importantly, MCAR T-2 cells proved to be safe in animal models, exhibiting reduced CRS and no significant toxicity while retaining effector functions. These cells achieved extended survival with tumor clearance lasting beyond 70 days, whereas mice treated with conventional CAR T cells succumbed by day 60.

Mechanistically, SG inhibits mTOR and increases cAMP, inducing autophagy by elevating cAMP levels, likely due to its canonical function on cAMP levels,^41^^,^^42^^,^^47^ which suppresses mTOR signaling, aligning with previous findings.^48^^,^^49^ Additionally, earlier studies have investigated the unconventional roles of GLP-1RAs, such as liraglutide, in inducing autophagy through mTOR modulation.^48^ Our findings demonstrate that KD of the GLP-1 receptor impairs autophagy induction and mitochondrial clearance during TR, emphasizing its critical role in supporting the maintenance of long-lived memory cells through metabolic reprogramming. Recently, GLP-1R expression has been observed in T lymphocytes,^50^^,^^51^^,^^52^ and, consistent with our results, it increases upon T cell activation.^53^ However, we found that GLP-1R antagonism enhanced CAR T cell anti-tumor activity, possibly due to differences in the models used. These findings highlight GLP-1R’s varied roles in immune responses and its potential impact on transcriptional and epigenetic reprogramming, warranting further investigation to understand CD8^+^ T cell exhaustion.

SG also upregulated key autophagy genes, suggesting that it influences autophagy beyond mTOR inhibition by promoting transcriptional changes. This dual effect likely contributes to prolonged CAR T cell persistence and memory differentiation. Recent studies emphasize the interplay between metabolic signaling and mitochondrial quality control in regulating mitophagy.^54^ Insulin receptor signaling, for instance, activates PINK1-mediated mitophagy.^55^ While we did not assess direct impact of cAMP on mTOR, it is known to inhibit mTORC1 via protein kinase A activation.^49^ Inhibiting mTOR shifts mitochondria to favor FAO, the preferred pathway for memory and stem-like T cells,^49^ consistent with our metabolic reprogramming results. mTOR KD in CAR T cells enhanced basal autophagy, reinforcing its critical role. These findings align with clinical studies linking SG and GLP-1 receptor agonists to weight loss and autophagy induction.^48^^,^^54^ KD of key autophagy genes like ATG14 or BECN1 replicated TR-induced CAR T cell dysfunction and reduced persistence, underscoring the importance of autophagy in maintaining CAR T cell functionality.

Recent studies emphasize the role of UrA in maintaining mitochondrial health by promoting mitophagy and biogenesis.^34^^,^^56^ UrA enhances CAR T cell activation, persistence, and effector functions via the ERK1/2-ULK1 pathway.^57^ Denk et al. identified Pink1-dependent mitophagy as a key mechanism driving T_scm_ cell expansion with UrA.^56^ However, prior studies have not examined the effect of UrA under TR conditions, crucial for assessing its long-term impact on CAR T cell function and persistence, nor have they detailed its mechanisms of action in CAR T cells. Our findings reveal that UrA alone does not sufficiently enhance CAR T cell function under TR but synergizes with GLP-1RA. Molecularly, GLP-1RA upregulates general autophagy gene expression, while UrA activates Atg4b, promoting LC3 maturation and clearing dysfunctional mitochondria, enhancing mitochondrial health. While GLP-1RA and UrA activate distinct pathways, they converge on the autophagy/mitophagy axis to synergistically improve CAR T cell functionality.

In conclusion, we identified the potential mechanism of targeting autophagy/mitophagy to enhance CAR T cell therapy. Using 3^rd^-generation CARs, which outperform 2^nd^-generation CARs in clinical outcomes,^58^^,^^59^ we demonstrated enhanced anti-tumor activity in lymphoma, leukemia, multiple myeloma (BCMA targeting), and brain cancer (SH-S5Y5 targeting) models. This strategy has broader potential in reprogramming CAR T cells for solid tumors, addressing their persistence challenges.^60^ Consistent with our findings, a recent study demonstrated that intercellular mitochondrial transfer to CD8^+^ T cells via nanotubes from bone marrow stromal cells enhances their metabolic fitness and anti-tumor efficacy—a process we have previously observed in lung epithelial cells.^61^^,^^62^ While these results are promising, further research is needed to translate these advancements into clinical applications.

### Limitations of the study

This study was conducted using *in vitro* and *in vivo* models, which may not entirely replicate the complexities of human tumors and immune responses. While GLP-1RA and UrA demonstrate potential in enhancing CAR T cell persistence and efficacy, their long-term safety and clinical effectiveness remain unverified. Additionally, the observed synergy between GLP-1R activation and autophagy/mitophagy pathways may vary across patient-specific factors and tumor microenvironments. Further research is required to validate these findings in diverse patient populations and tumor types.

## Resource availability

### Lead contact

Further information and requests for resources and reagents should be directed to and will be fulfilled by the lead contact, Dr. Tanveer Ahmad, tahmad7@jmi.ac.in.

### Materials availability

CAR generated in this study will be made available from Dr. Tanveer Ahmad, tahmad7@jmi.ac.in, on request, but we may require a payment and/or a completed Materials Transfer Agreement if there is potential for commercial application.

### Data and code availability

• The data supporting the findings of this study are available from the lead contact (Dr. Tanveer Ahmad, tahmad7@jmi.ac.in) upon request. Any additional datasets generated during the study will be made available upon request.
• No custom computer code was used in this study.
• Any additional information required to reanalyze the data reported in this paper is available from the lead contact (Dr. Tanveer Ahmad, tahmad7@jmi.ac.in) upon request.

## Acknowledgments

The authors would like to sincerely thank Dr. Sivaprakash (CSIR-IGIB) for his insightful contributions and discussions during the project’s execution. The authors also acknowledge the valuable input provided by Mohd Zama Ansari, Manish Kumar, and Jyotirmoi Aich during the manuscript preparation. T.A. expresses his gratitude for the financial assistance provided through grant from the Department of Biotechnology (DBT), India (BT/PR40619/MED/30/2294/2020). S.A. extends her appreciation to 10.13039/501100006261Taif University, Saudi Arabia, for supporting this work through project number TU-DSPP-2024-23.

## Author contributions

A.A., M.S., M.S.A., D., M.I.F., V.C., A.S., R.A., I.A., S. Sharma, M.P., I.M.U., U.B., S.N.S., R.G., and R.C. performed the experiments and analyzed the data. A.A. and T.A. designed the study and wrote the manuscript. S.A., S. Sagar, V.P.S., G.K., A.K.S., U.M., S.S.R., and I.R. provided technical support and helped in data analysis. All authors approved the final version of the manuscript.

## Declaration of interests

T.A. and A.A. have filed a patent for their work titled “Method for Enhancing CAR T Immunotherapy Through Metabolic Engineering.”

## STAR★Methods

### Key resources table

**Table:** 

REAGENT or RESOURCE	SOURCE	IDENTIFIER
**Antibodies**		

Anti-Biotin-PE (Biotin Antibody, PE, REAfinity)	Miltenyi Biotech	Cat#130-111-068
CAR-Detection antibody	Miltenyi Biotech	Cat#130-129-550; RRID: AB_2811310
Anti-GLP-1R antibody	Thermo Fisher Scientific	Cat#PA5-97789; RRID: AB_2812404
CD45RO antibody APC	BD Biosciences	Cat#340438
CD45RO antibody	BD Biosciences	Cat#562641; RRID: AB_2737696
CCR7 antibody	BD Biosciences	Cat#567313
CCR7 antibody	BD Biosciences	Cat#565868; RRID: AB_2744304
CCR7 antibody	BD Biosciences	Cat#552176; RRID: AB_394354
PD-1 antibody	BioLegend	Cat#367408; RRID: AB_2566678
PD-1 antibody	BD Biosciences	Cat#567617
Anti-CD19 antibody	BD Biosciences	Cat#345777; RRID: AB_2869004
Anti-CD8 antibody	BD Biosciences	Cat#557945; RRID: AB_396953
Anti-Granzyme B antibody	BD Biosciences	Cat#560211; RRID: AB_1645488
Anti-Granzyme B antibody	BD Biosciences	Cat#561142; RRID: AB_10561690
Anti-Perforin antibody	BD Biosciences	Cat#563393; RRID: AB_2738178
Anti-Perforin antibody	BD Biosciences	Cat#567722
Anti-Perforin antibody	BD Biosciences	Cat#563763; RRID: AB_2738410
Anti-LAG-3 antibody	BioLegend	Cat#369318; RRID: AB_2715781
Anti-Atg14 antibody	Abcam	Cat#ab315009; RRID: AB_2890490
Anti-Beclin-1 antibody	Cell Signaling Technology	Cat#3738; RRID: AB_490837
Anti-LC3-II antibody	Cell Signaling Technology	Cat#2775; RRID: AB_915950
GAPDH antibody	CST	Cat#2118; RRID: AB_561053
mTOR antibody	CST	Cat#2972; RRID: AB_330978
Anti-TFAM antibody	CST	Cat#8076; RRID: AB_10949110
TOM20 antibody	Abcam	Cat#ab56783; RRID: AB_945896
Anti-p62 antibody	Cell Signaling Technology	Cat#5114; RRID:AB_10624872
Anti-human CD4 antibody	BD Biosciences	Cat#555349; RRID: AB_398593
Anti-human CD3 antibody	BD Biosciences	Cat#564465; RRID: AB_2744386
IFN-Y	BD Biosciences	Cat#562017; RRID: AB_398580
IL-4	BD Biosciences	Cat#554484; RRID: AB_395423
IL-17	BD Pharmingen	Cat#569262
CD25	BD Biosciences	Cat#567214
FoxP3	BD Pharmingen	Cat#560887; RRID: AB_1645349
PD-1	BD Biosciences	Cat#563789; RRID: AB 2738956
LAG-3	BD Biosciences	Cat#565720; RRID: AB 2869657
Goat Anti-Rabbit IgG H&L (Alexa Fluor® 488)	Abcam	Cat#ab150077
Goat Anti-Rabbit IgG H&L (Alexa Fluor® 594)	Abcam	Cat#ab150080

**Bacterial and virus strains**		

pMD2.G (VSV-G envelope)	Addgene	Cat# 12259
psPAX2	Addgene	Cat# 12260
pRL-SV40P	Addgene	Cat# 27163

**Biological samples**		

Human Peripheral blood mononuclear cells	Apollo Indraprastha Hospital, New Delhi	N/A

**Chemicals, peptides, and recombinant proteins**		

Seahorse Real-Time Cell Metabolic Analysis reagents	Agilent	103771–100
FastSelect reagent (for mRNA enrichment)	Qiagen	334222
DAPI	Invitrogen	P36966
SYTOX Green dye	Thermo Fisher Scientific	S7020
IVISbrite™ D-Luciferin	PerkinElmer	122799
MitoTracker Green (for mitochondrial DNA content)	Invitrogen	M7514
Human recombinant IL-2	Miltenyi Biotech	130-097-743
Anti-CD3/CD28 antibody (T cell TransAct)	Miltenyi Biotech	130-111-160
RIPA cell lysis reagent	Thermo Fisher Scientific	89900
DMEM (Dulbecco’s Modified Eagle Medium)	Gibco	11965092
FBS (Fetal Bovine Serum)	Gibco	10270106
PEIpro (Polyethylenimine)	Polyplus	101000033
OptiMEM	Gibco	31985070
LentiX (concentrating reagent)	Takara	631232
Histamine	Sigma	Y0001779
CFSE (Carboxyfluorescein succinimidyl ester)	Invitrogen	C34554
MitoSOX Red	Invitrogen	M36008
TMRE (Tetramethylrhodamine, Ethyl Ester)	Invitrogen	T669
Lyso Tracker Deep Red	Thermo Fisher Scientific	L12492
FCCP (Carbonyl cyanide 4-(trifluoromethoxy)phenylhydrazone)	MERCK	C2920-10MG
7-AAD	Thermo Fisher Scientific	A1310

**Critical commercial assays**		

K-LISA™ mTOR Activity Kit	Sigma-Aldrich	CBA104
Cyto-ID Autophagy detection kit	Enzo Life Sciences	ENZ-51031-K200
IVISbrite™ D-Luciferin kit	Revvity	770504
Calcein cobalt assay	Thermo Fisher Scientific	M34153
Human GLP1 (7–36) ELISA Kit	Abcam	ab184857
cAMP-Glo™ Assay	Promega	V1501

**Deposited data**		

scRNA-seq data for patient CAR T cells	Good et al.^32^	GEO: GSE168940
Bulk RNA-seq	This study	https://doi.org/10.5281/zenodo.14725625
Flow cytometry	This study	https://doi.org/10.5281/zenodo.14725625

**Experimental models: Cell lines**		

HEK-293T	ATCC	CRL-3216
Raji	ATCC	CCL-86
NALM6	ATCC	CRL-2266
MM.1S	ATCC	CRL-2974
SH-SY5Y	ATCC	CRL-2266
Daudi	ATCC	CCL-213
Jurkat	ATCC	TIB-152
Raji-mCherry-luciferase	N/A	This paper
NALM6-mCherry-luciferase	N/A	This paper
SHS-5Y5-mCherry-luciferase	N/A	This paper
MM1.S mCherry-luciferase	N/A	This paper
Jurkat mitophagy reporter	N/A	This paper
Jurkat autophagy reporter	N/A	This paper

**Experimental models: Organisms/strains**		

NOD.Cg-Prkdcˆscid Il2rgˆtm1Wjl/SzJ	The Jackson Laboratory	005557
C.B.Igh-1b/GbmsTac-PrkdcˆscidLystˆbgN7	Taconic Biosciences	491

**Oligonucleotides**		

BECN1 shRNA	This paper	Table S6
ATG14 shRNA	This paper	Table S6
GLP-1R shRNA	This paper	Table S6
mTOR shRNA	Sarbassov et al.^63^	Addgene #1855
BECN1 Primers:	This paper	Table S6
ATG14 Primers	This paper	Table S6
GLP-1R Primers	This paper	Table S6
CD19 ScFv Primers	This paper	Table S6
mTOR Primers	This paper	Table S6

**Recombinant DNA**		

pCLBW cox8 EGFP mCherry	Rojansky et al.^31^	Addgene #78520
pCDH-EF1a-mCherry-EGFP-LC3B	Wulansari et al.^64^	Addgene #170446
LC3-mCherry (LC3-mCherry plasmid)	This Paper	N/A
pLYS1-FLAG-MitoGFP-HA	Sancak et al.^65^	Addgene #50057
CD19 CAR scFv sequence plasmid	This Paper	N/A
GLP-1 secreting CAR plasmid	This Paper	N/A
shRNA encoding BECN1, ATG14, and GLP-1R plasmids	This Paper	N/A
mCherry-Luciferase plasmids	This Paper	N/A
pRSV-Rev	Dull et al.^66^	Addgene #12253
pMDLg/pRRE	Dull et al.^66^	Addgene #12251
pMD2.G	Didier Trono Lab	Addgene #12259
pCDH-EF1a-cox8-EGFP-mCherry	This Paper	N/A
pMOS008: GCaMP6F calcium sensor (cytosolic)	Werley et al.^67^	Addgene #163045
pCAG mito-RCaMP1h	Hirabayashi et al.^68^	Addgene #105013
pC013-Twinstrep-SUMO-huLwCas13a	Gootenberg et al.^69^	Addgene #90097
pGEX-4T1_ATG4B_BamHI_NotI	Fracchiolla et al.^70^	Addgene #190862

**Software and algorithms**		

FlowJo™ Software	BD Biosciences	https://www.flowjo.com/solutions/flowjo
GraphPad Prism version 9	GraphPad Software	https://www.graphpad.com
ImageJ	National Institutes of Health	https://imagej.nih.gov/ij
nf-core pipelines	N/A	https://nf-co.re
KEGG (Kyoto Encyclopedia of Genes and Genomes)	KEGG	https://www.genome.jp/kegg/
ShinyGO v0.61	South Dakota State University Bioinformatics	https://bioinformatics.sdstate.edu/go/
Seurat	Satija Lab	https://satijalab.org/seurat/
ggplot2	ggplot2	https://ggplot2.tidyverse.org/
AutoDock Vina	Version 1.1.2	https://vina.scripps.edu/
CASTp (Computed Atlas of Surface Topography of Proteins)	CASTp	http://sts.bioe.uic.edu/castp/index.html?2cpk
frustratometeR	Parra et al.^71^	https://frustratometer.qb.fcen.uba.ar/
VICTOR Nivo Multimode Microplate Reader	Revvity	https://www.revvity.com
IVIS Lumina LT *In Vivo* Imaging System	Revvity	https://www.revvity.com
iGeak_RNA-seq software	iGeak	https://github.com/omicsCore/iGeak
Schematic illustrations	Bio render	https://www.biorender.com

**Other**		

QIAGEN Pure mRNA beads	QIAGEN	180244
Protein A/G Sepharose beads	Abcam	ab193262

### Experimental model and study participant details

#### Cell line details

The cell lines HEK-293T, Raji, NALM6, MM1, SH-SY5Y, and Daudi were purchased from the American Type Culture Collection (ATCC) and cultured following ATCC-recommended protocols. HEK-293T-cells were maintained in DMEM with 10% FBS at 37°C, 5% CO_2_, and 95% humidity, with sub culturing at 70–80% confluency using trypsin. Raji, NALM6, MM1, and Daudi cells were cultured in RPMI-1640 medium with 10% FBS under similar conditions, with Raji and Daudi cells sub-cultured at 2–3 × 10^6^ cells/mL and NALM6 at 1–2 × 10^6^ cells/mL, while MM1 cells were split every 2–3-day. SH-SY5Y cells were grown in DMEM/F-12 (1:1) with 10% FBS under the same conditions, passaged at 70–80% confluency using trypsin.

#### Human samples

Peripheral blood mononuclear cells (PBMCs) were obtained from healthy adult donors between 25 and 50 years of age, with 30 male and 25 female samples obtained at Apollo Indraprastha Hospital, New Delhi, in accordance with protocols approved by the Institutional Review Board. Additionally, PBMCs were collected from three patients’ age; 28 years, male; 36 years, male, and 47 years, female, diagnosed with B-ALL, following written informed consent (Document S2) and adherence to the hospital’s ethical and regulatory standards. The procedures were overseen by Dr. Gaurav Kharya, Senior Consultant in Pediatric Hematology Oncology & Immunology at Indraprastha Apollo Hospital, New Delhi. The blood samples utilized were surplus specimens that would otherwise have been discarded after marker analysis.

#### Animal experiments

NSG (NOD.Cg-Prkdcˆscid Il2rgˆtm1Wjl/SzJ) and NCG (NOD-Prkdcem26Cd52Il2rgem26Cd22/NjuCrl), aged 6–8 weeks, were purchased from The Jackson Laboratory and Charles River Laboratories, respectively and housed in the Laboratory. The mice were maintained on a 12 h light/dark cycle at 22°C with *ad libitum* access to food and water. Additionally, 4–6-week-old female SCID-beige mice (strain C.B.Igh-1b/GbmsTac-PrkdcˆscidLystˆbgN7) were sourced from Taconic Biosciences for CRS studies. These animals were also kept under a 12 h light/dark cycle at 22°C with free access to food and water. All animal experiments were conducted following approval from the respective Ethics Committee for Animal Experiments. All animal experiments were approved by Institutional Animal Ethics Committee, MicroCRISPR Pvt Ltd; CSIR-Institute of Chemical Biology and CSIR-Institute of Genomics and Integrative Biology.

### Method details

#### Cell lines

Reporter stable cell lines for Raji, NALM6, MM1, SH-S5Y5 and Daudi were generated through transduction with an LV plasmid containing mCherry and luciferase reporters. Subsequently, the cells were sorted using mCherry via FACS. Expansion of single-cell clones derived from the sorted cells was carried out to ensure consistency and homogeneity of the clones. Jurkat T cell line for drug screening was obtained from ATCC and cultured in the RPMI media as recommended. CHO cell lines were obtained from ATCC and cultured as recommended.

#### Plasmid design and lentivirus production

The CD19 CAR, BCMA CAR and GD2 CAR plasmid used in this study was generated by cloning an in-house designed synthetic fragment obtained from GenScript into our lentiviral vector backbone. The CD19 CAR sequence is made up of scfv derived from FMC63 clone, CD8 hinge and CD8 transmembrane domain along with CD28 and 4-1BB co-stimulatory domains and CD3ζ domain. The BCMA CAR (clone: 4C8A) and GD2 CAR (Clone: 14G2a) were similarly cloned in the above configuration. Similarly, nGLP-1 secreting CARs were generated using a synthetic gene fragment obtained from GenScript and cloned in this CAR Plasmid.

The mitophagy reporter was generated by PCR amplification of the Cox8 EGFP mCherry from pCLBW Cox8 EGFP mCherry (Addgene #78520) followed by cloning in our in-house LV vector backbone. Similarly, autophagy reporter was generated by cloning pMRX-IP-GFP-LC3-RFP-LC3ΔG (Addgene #84572) into the LV vector backbone. LC3-mCherry plasmid was made by cloning the LC3-mCherry synthetic fragment obtained from GenScript in the LV backbone. LC3 GFP plasmid was obtained from Addgene (Plasmid #11546) and cloned into the LV backbone. Mitochondrial target GFP plasmid (pLYS1-FLAG-MitoGFP-HA (Plasmid #50057)) was generated as mentioned previously.^72^ shRNA sequences of BECN1, ATG14, GLP-1R were designed using shRNA Design Tool (VectorBuilder) and the sequences were cloned as described by us previously.^72^ To generate the reporter tumor cell lines like Raji, SH-SY5Y, MM.1S, and NALM6, mCherry-Luciferase plasmids were used. These plasmids were generated by obtaining the mCherry and luciferase as synthetic fragments and cloned in the in-house LV backbone vector. The shRNA plasmid for mTOR was obtained from Addgene and used in the study (Plasmid #1855).

HEK-293T cells were plated in T-175 at a density of 4.2 × 10^6^ cells in 30 mL of DMEM supplemented with 10% FBS. Following 48 h of cell seeding, the cells were transfected with third-generation lentiviral system containing plasmids: pRSV-Rev, pRRE, pMD2.G/VSVG and our third generation CD19 CAR transgene plasmid incorporating CD28 and 4-1BB co-stimulatory domains (design patented). PEIpro (Polyplus) was used as the transfection reagent, and the cells were maintained in the transfection medium for 48 h initially, followed by an additional 24 h. Supernatants were harvested at 48 h and 72 h post-transfection following a media change. The collected supernatant was concentrated using LentiX (Takara), and the resulting pellet was resuspended in 600μL of OptiMEM and aliquots were made before storing at −80°C for subsequent titer determination. Transduction efficiency was assessed through serial dilution of the concentrated lentiviral particles (LV). Similarly, LV particles were generated for autophagy reporter, mitophagy reporter, shRNA encoding GLP-1R, ATG14 and BECN1 using the respective transgene plasmids encoding these genes. The genes from respective plasmids (obtained from Addgene) were PCR amplified and sub-cloned into the lentiviral backbone (pLYS1-FLAG-MitoGFP-HA) using Nhe1 and BsrG1 sites. For shRNA mediated knockdown experiments, a set of 3–4 different shRNAs were evaluated (key resources table). The list of plasmids used is given in the key resources table.

#### shRNA mediated knockdown

CAR T cells were transduced with the shRNA encoding LV particles at the multiplicity of infection (MOI) of 5. The LV transduction was done along with the CAR transduction at day1 after the T cell isolation at day 0. The shRNA mediated knockdown was evaluated by using RT-qPCR or immunoblotting as described by us previously.^72^

#### Primary cell isolation and transduction

The PBMCs were extracted from whole blood samples by density gradient centrifugation using Ficoll-Paque (Cytiva) as per the manufacturer’s guidelines. Subsequently, CD4/CD8 magnetic beads were used to isolate T-cells. Similarly, CD4 and CD8 magnetic beads (Miltenyi Biotec) were used to isolate pure CD4^+^ and CD8^+^ T cell populations. These T cells were then activated using anti-CD3/CD28 stimulation (T cell TransAct, Miltenyi Biotec) and cultured in TexMACS T cell culture medium supplemented with 100 IU/mL of human recombinant IL-7 and IL-15 (Miltenyi Biotec). Following 24 h of activation, the T-cells were transduced with lentiviral concentrate at MOI of 5 and incubated for an additional 48 h before media change. Fresh medium was replenished every 2-day throughout the experiment. The percentage of transduced T cells was determined using the CD19 CAR detection reagent (Miltenyi Biotec) following the manufacturer’s instructions, and flow cytometry analysis was done after day 5 of transduction, unless otherwise specified.

Patient blood samples were processed to isolate PBMCs using Ficoll density gradient centrifugation. PBMCs were further separated into T cells and B cells. For T cell isolation, standard procedures were followed, while for B cell isolation, magnetic beads conjugated to CD19 antibodies (Miltenyi Biotech) were utilized. The isolated B cells were confirmed via flow cytometry using CD19-specific antibodies. After isolation, the cells were cultured in RPMI medium supplemented with 10% FBS and subsequently co-cultured with CAR T cells, which were generated from the patient-derived T cells. All procedures adhered strictly to ethical guidelines and regulatory standards to ensure patient safety and data integrity.

#### CAR T cell proliferation, persistence and cell death

CAR T cell proliferation assay was done using CFSE (Carboxyfluorescein succinimidyl ester; CellTrace CFSE - Cell Proliferation Kit; Thermo Fisher Scientific). CAR T cells were stained with CFSE dye and cell proliferation was tracked at various time intervals (Day-3, 5, 7, 9, 11, 13, 15, 17 and 19 and as mentioned). Initially, CAR T-cells are harvested and washed to ensure cell viability and purity. The cells were then resuspended in CFSE solution at an appropriate concentration (10uM) and incubated for a specific period of 2-day before the first analysis using flow cytometry. The fluorescence intensity of CFSE decreases with each round of cell division, enabling quantification of cell proliferation and assessment of CAR T cell proliferation dynamics over the specified time period.

CAR T cell persistence was measured by counting the cell number at day-0 before co-culture and at day 7, 13, 21 and 28 after the co-culture with Raji cells. The co-culture was done at day, 6, 8, 10, 12, 14, 16, 18 and 20. The initial number of CAR T cells used for was 5x10^4^ and the Raji cells were used at a number of 1x10^4^, added at the indicated time intervals. The cell count was done by automated cell counter (Countess 3 FL Automated Cell Counter) and plotted as the number of CAR T cell persistence. The left over Raji cells were detected by fluorescence detector and subtracted from the total cell number.

Cell death analysis was conducted using SYTOX Green dye, followed by acquisition via flow cytometry, as previously described by us.^72^ The data was presented either as the representative flow cytometry histograms indicating percentage mean fluorescence intensity of SYTOX Green or a percentage cell death using bar graphs. Live-cell imaging was performed using the MuviCyte Live-Cell Imaging system (Revvity). Raji cells were co-cultured with CAR T cells at a 1:5 ratio for 12 h, followed by an additional 12 h of live-cell imaging. Prior to the co-culture, CAR T-cells were cultured in the presence of SG + UrA for 7-day.

#### Drug screening

We developed Jurkat reporter cell lines to monitor autophagy and mitophagy for drug screening purposes. The mitophagy reporter line was created using a plasmid that includes the Cox8 mitochondrial targeting sequence fused with GFP and mCherry at the N-terminus, following the method outlined by Rojansky et al. (2016).^31^ This cell line expresses a construct where, under normal conditions, mitochondria emit both green and red fluorescence, appearing yellow. However, when mitophagy is induced, mitochondria within acidic compartments show increased red fluorescence and reduced GFP fluorescence, as GFP is selectively quenched in the low pH environment of the autophagosome. Similarly, the autophagy reporter cell line was generated by stably transducing the GFP-LC3-RFP construct. We engineered lentiviral vectors to deliver either the mitophagy reporter plasmid (mCherry-Cox-8-EGFP) or the autophagy reporter plasmid (GFP-LC3-RFP), both based on a similar mechanism. In the autophagy reporter plasmid, upon autophagy induction, the LC3-GFP-RFP complex is taken into autophagosomes, where the acidic pH quenches GFP but not RFP. These vectors were used to transduce Jurkat cells, and fluorescence-activated cell sorting (FACS) was employed to isolate GFP-positive cells. These sorted Jurkat cells were then treated with a library of 27 validated compounds related to autophagy, mitophagy, and mitochondrial metabolism. The drug screening involved testing these compounds individually, in pairs, or in triple combinations, resulting in a total of 3,357 combinations. Following concentrations were used for each compound; Acarbose: 1 μM, 5 μM, 10 μM; Brefeldin A: 0.1 μM, 1 μM, 5 μM; Canagliflozin: 1 μM, 5 μM, 10 μM; Curcumin: 5 μM, 10 μM, 20 μM; Dapagliflozin: 1 μM, 5 μM, 10 μM; Epitalon: 10 nM, 100 nM, 500 nM; Hydralazine: 0.1 μM, 1 μM, 5 μM; Melatonin: 5 μM, 10 μM, 50 μM; Metformin: 10 μM, 100 μM, 1000 μM; MitoTEMPO: 1 μM, 5 μM, 10 μM; N-acetyl cysteine: 10 μM, 100 μM, 1000 μM; NMN: 100 μM, 250 μM, 500 μM; PQQ: 1 μM, 10 μM, 20 μM; Rapamycin: 0.1 nM, 1 nM, 10 nM; Resveratrol: 1 μM, 2.5 μM, 5 μM; Rosiglitazone: 5 μM, 10 μM, 20 μM; Semaglutide: 1 nM, 5 nM, 10 nM; Spermidine: 1 μM, 5 μM, 10 μM; Spautin-1: 100 nM, 250 nM, 500 nM; Simvastatin: 1 μM, 5 μM, 10 μM; Thapsigargin: 100 nM, 500 nM, 1000 nM; Torin 1: 100 nM, 250 nM, 500 nM; Torin 2: 100 nM, 250 nM, 500 nM; Trehalose: 1 mM, 10 mM, 20 mM; Urolithin A: 10 μM, 20 μM, 50 μM; Verapamil: 1 μM, 5 μM, 10 μM; Zidovudine: 1 μM, 10 μM, 20 μM. Carbonyl cyanide p-(trifluoromethoxy) phenylhydrazone (FCCP) was used at a concentration of 1 μM. The cells were plated at a density of 1 x 10^4^ cells per well in a 96-well plate and cultured for 24 h before the assay was performed. Fluorescence measurements were performed using the VICTOR Nivo Multimode Microplate Reader (Revvity), with excitation for GFP at 488 nm and emission set at 520 nm. For RFP, the excitation was set at 555 nm and emission at 580 nm, and for mCherry, the excitation was set at 580 nm with emission at 610 nm. FCCP was used as a positive control for autophagy/mitophagy induction. The extent of mitophagy or autophagy was assessed by calculating the ratio of mCherry/GFP or RFP/GFP signals respectively and presented as a heatmap of the ratios. The top five combinations of drugs were selected for further assessment of mitophagy, cell death, and cell proliferation in primary CAR T cells.

#### Flow cytometry

Flow cytometry analyses were conducted using the FACS Aria, FACS melody, FACS Accuri and FACS Lyric system from BD Biosciences, and the resulting data was processed using FlowJo software. To assess CAR expression, 1 x 10^6^ cells were first stained with CD19 CAR detection antibody, followed by a 10 min incubation and subsequent washing with 1 mL of FACS buffer. This was followed by a 10 min incubation with a secondary antibody, Anti-Biotin-PE (Biotin Antibody, PE, REAfinity; Miltenyi Biotec). The stained cells were then washed again and resuspended in 500ul FACS Buffer and analyzed using flow cytometer. The findings are presented as the percentage of CD19 CAR transduction efficiency relative to the un-transduced cells.

For T cell immunophenotyping, 1 x 10^6^ cells were stained with respective antibodies and incubated for 30 min on ice, in dark, followed by washing with 1X PBS two times, then acquisition and analysis by flow cytometry. The antibody panels which were used are included in the key resources table. All the antibodies were either directly fluorophore conjugated, or fluorescent conjugated secondary antibodies were used. Flow cytometry was also employed to analyze Granzyme B and Perforin, PD-1 and LAG-3 levels. T cell polarization was done in the isolated CD4^+^ T cells and cultured in presence of SG + UrA for 7-day before the flow cytometry analysis. Th1 cells were identified by intracellular staining of cells with IFN-γ, Th2 was identified by IL-4, Th17 by IL-17 and Treg by staining with CD25 and Foxp3. Cell death analysis was done by staining the cells with SYTOX green dye followed by acquisition using flow cytometry as described by us previously.^72^ GLP-1R expression was determined by flow cytometry in CAR T cells on day 3 post-activation with CD3/CD28. Further, the expression of GLP-1R was assessed in different T cell subsets on day 7 post-activation. Cells were labeled with anti-GLP-1R antibody in conjunction with antibodies targeting CD45RO, CCR7, and PD-1 to distinguish T cell subsets including T naive, T stem cell memory, T central memory, T effector memory, and T exhaustion. Atg14 and Beclin-1 were stained for 20 min in respective primary antibodies. Following 3 washing steps, a secondary antibody conjugated with either Alexa Fluor 488 or PE were used, respectively. Notably, 7-AAD was used to gate on the live/dead cell population during the flow cytometry analysis. The data was analyzed by FlowJo and presented as the mean fluorescence intensity (MFI). MFI values were calculated based on all evaluated cells. Respective isotype controls were used for the flow cytometry analysis (key resources table).

#### CAR T cell autophagy and mitophagy reporters

CAR T cells were transduced with autophagy or mitophagy reporter lentiviruses, following the procedure detailed earlier. This transduction was done on day 5 post-CAR T transduction, and subsequent assays were conducted on days-7, 15, and 21 unless otherwise indicated. Flow cytometry was employed to acquire the data, and FlowJo software was utilized for analysis. The degree of mitophagy or autophagy was evaluated by determining the ratio of mCherry/GFP or RFP/GFP signals and was depicted through representative dot plots and histograms generated from three separate experiments.

#### Autophagy and mitophagy assays

Mitophagy assessment was conducted using two distinct techniques. In the first method, cells were labeled with MitoTracker Green (MTG) (Thermo Fisher Scientific) to label mitochondria and Lysotracker Deep Red (LTDR) (Thermo Fisher Scientific) to label lysosomes, followed by fluorescence measurement using the VICTOR Nivo Multimode Microplate Reader (Revvity) to determine the change in the fluorescence of MTG relative to LTDR. The staining protocol followed our previously described method.^73^ Upon mitophagy induction, the MTG signal decreases, while the LTDR signal remains largely constant. The mitophagy rate was represented as the ratio of change in fluorescence from green toward red. FCCP was employed to induce mitophagy in this assay, and the readings were taken at 0 min and after 60 min to monitor the change in fluorescence, following our established protocol.^73^ The second approach involved transducing cells with a mitophagy reporter, as mentioned above, followed by flow cytometry analysis for GFP and mCherry signals. The signal was obtained by flow cytometry, and the data were presented as the ratio of mCherry to GFP and analyzed using FlowJo.

For autophagy analysis, two methods were utilized. In the first method, cells were stained with the CYTO-ID Autophagy Detection Kit from Enzo Life Sciences, following the manufacturer’s instructions. The signal was obtained by flow cytometry, and the data were presented as the ratio of mCherry/GFP and analyzed using FlowJo. Additionally, an autophagy reporter plasmid encoding GFP and RFP LC3 was used to assess autophagy as mentioned above. Autophagosome formation was evaluated by transducing CAR T cells with LC3-mCherry and imaging with a Nikon Ti2E confocal microscope. Subsequent image analysis was conducted using ImageJ software to determine integrated density, as per our previous work.^72^^,^^73^ Furthermore, autophagy-related protein expression was assessed via flow cytometry using antibodies against specific autophagy-related proteins such as Atg14, Beclin-1, and p62. The data from these experiments are presented as representative FACS histograms and bar graphs from three independent trials.

#### LC3II FRET-assay

We constructed the FRET-LC3II plasmid by inserting CFP near the N terminus and YFP at the C terminus of the LC3-mCherry plasmid, after removing the mCherry component. For expression in a bacterial system, the CFP-LC3-YFP insert was PCR amplified and cloned into the pC013-Twinstrep-SUMO-huLwCas13a vector (Plasmid #90097) according to our previously established protocol.^74^ The Atg4b protein was sourced from Addgene (Plasmid #190862), and recombinant production was conducted in E. coli cells. Based on our previously established protocol, we purified Atg4B protein at a concentration of 250 μg/mL and incubated with FRET-LC3B at 37°C for 0, 5, or 15 min. Drug treatments of SG (5 nM, nGLP-1 (5nm) or UrA (10 μM) done at 0 min. The assay was conducted in a 96-well plate, maintaining a reaction volume of 200 μL. The cleavage of LC3B substrates was assessed using the VICTOR Nivo Multimode Microplate Reader (Revvity). Excitation was set at 430 nm, and emissions were measured at 475 nm (CFP) and 530 nm (YFP). The RFU ratio of 530 nm–475 nm was calculated to quantify the cleavage extent, with the ratio decreasing proportionally to substrate cleavage, based on previous study.^33^ The data are presented as the percentage ratio of relative fluorescence units (RFU) at 530 nm–475 nm.

#### Immunofluorescence

For the GLP-1R immunofluorescence assay, CAR T-cells were plated at a density of 1 x 10^6^ cells on day 3 post-activation with CD3/CD28 and subjected to immunostaining. The cells were stained with a GLP-1R rabbit primary antibody at a dilution of 1:1000 with a 30 min incubation on ice. Following this, the cells were treated with anti-rabbit Alexa Fluor 568 for 20 min on ice, followed by three washes with 1XPBS. After pelleting, the cells were fixed using 1% paraformaldehyde, mounted with a coverslip, and visualized through confocal microscopy (Leica), following our previously established protocol. DAPI was included in the mountant to label the nuclei.

Mitochondrial DNA content was measured by staining the cells with anti-TFAM antibody. Similarly, mitochondrial morphology was assessed by staining the cells with TOM20 following the protocol as described by us previously. The mitochondrial shape analysis in CAR T-cells was done as per our previously established method of mitochondrial shape classification.^75^

Mitochondrial permeability transition pore (mPTP) formation assay was measured as described by us previously using Calcein cobalt assay.^72^

LC3 GFP imaging was done in CAR T cells transduced with the LV encoding LC3 plasmid following treatment with SG, UrA or a combination of SG + UrA. The images were quantitated using ImageJ by specifically focusing the LC3 GFP puncta formation upon induction with the drugs. The data is represented as the number of LC3 puncta counted per cell.

#### Immunoblotting and RT-qPCR analysis

Similarly, the immunoblotting procedure and RT-qPCR were conducted following the protocols outlined in our previous publications.^72^^,^^73^ Briefly, anti-Atg14, Beclin-1, and LC3-II antibodies were used at a dilution of 1:1000, followed by incubation with an HRP-conjugated secondary antibody at a dilution of 1:5000. GAPDH antibody was used at a dilution of 1:2000. Images were captured, signal quantification was performed, and the data was presented as the expression levels of the respective signals as described previously.^72^^,^^73^

RT-qPCR analysis was done after total RNA extraction and subsequent cDNA synthesis. This analysis was conducted on CAR T cells that were transduced with non-targeting control (Scram) and shRNA targeting GLP-1R, BECN1, and ATG14, respectively. The procedures for analysis and representation of the signal were carried out following our previously established methodology.^73^ CAR copy number was determined by the PCR of the genomic DNA obtained from the CAR T cells using CD19 scFv specific primers as well as LV primers using the Lenti-X Provirus Quantitation Kit (Takara). The list of primers is given in key resources table.

#### *In vitro* tumor re-challenge

For the TR experiment, cells were initially seeded in a 48-well plate at a ratio of 1:5 target to effector cells (Raji:CAR T). The TR started on day 5 after CAR Transduction. Subsequently, every 2-day, the cells were split, and the supernatant was preserved for further analysis. The divided cells were then separated into two portions: one-half was used for ongoing re-challenge with additional Raji cells, while the other half underwent flow cytometry analysis to assess the CAR T cell count, reflecting CAR T cell proliferation over time. This TR process was continued for 28-day post-CAR transduction, unless stated otherwise. The protocol for TR involved harvesting and adding Raji cells for TR in designated wells, followed by subsequent harvests at various time points post-TR. Flow cytometry analysis was conducted every 2-day, and the resulting data was depicted as the rate of CAR T cell proliferation relative to the initial seeding cell density.

#### ELISA

The ELISA assay for cytokines such as IFN-γ, IL-2, IL-6, and TNF-α was performed using the total cell protein isolated from the hCD3 cells in TR mice. The total cell protein (TCP) was isolated using RIPA cell lysis reagent as described by us previously.^73^ For Raji cell lymphoma model (as shown in Figure 7A), CAR T cells were isolated at day 14 after Raji cell administration and analysis was performed. The isolated CAR T cells were co-cultured with Raji cells for 24 h and the supernatant was collected for analysis. Similarly, for *in vitro* experiments, supernatant from the co-cultured Raji cells with CAR T cells for 24 h was collected and used for the assay. A volume of 100μL of TCP or supernatant collected was utilized for cytokine analysis. The assay was conducted at different time points following co-culture, or if mentioned as a single time point, the sample was collected after 21-days of co-culture for analysis. Similarly, ELISA assay was performed with mouse ELISA kits for IL-6, GMCSF, CCL2, IL-1 β and TNF-α. The ELISA assay was performed using 50 μL of serum and the protocol was described by us previously. Data collection was carried out using the VICTOR Nivo Multimode Microplate Reader (PerkinElmer), and data analysis was performed using GraphPad Prism software. The results were presented as the signal in picogram/mL of the supernatant, following our previously established protocol.^73^ Similarly, GLP-1 levels were measured in the cell culture supernatant after the initial screening measured secreted GLP-1 in CAR T cell supernatants at day 7 post-transduction (as shown in Figure 6B). The assay was performed using Human GLP-1 (7–36) ELISA Kit (Abcam) as per the recommended protocol. Briefly, 100μL of the supernatant was used for the assay. Similarly, GLP-1 levels were measured in 25 μL of serum prepared from the peripheral blood of the mice (as shown in Figure 7G).

#### Cytotoxicity assays

To assess the cytotoxic efficacy of CD19-targeted CAR T cells and scGLP-1-CAR T cells, cultured with or without UrA, SG or their combination as per the experimental conditions, the cells were co-cultured with Raji, Nalm-6, or Daudi cells for a duration of 24 h. This co-culture was done across various target to effector ratios (1:1.25; 1:2.5; 1:5; 1:10) in a 96-well flat-bottom plate. The cells were seeded in either triplicates or quadruplets for the experiment. Subsequent to a 24 h co-culture period, luciferase activity was assessed using the IVISbrite D-Luciferin kit following the manufacturer’s instructions (PerkinElmer).

#### Mitochondrial assays

Mitochondrial assessments were conducted through several methods as described by us previously.^72^^,^^73^ Mitochondrial reactive oxygen species (mtROS) levels were evaluated by staining cells with MitoSOX Red. The mitochondrial membrane potential was assessed by staining cells with TMRE. Mitophagy rates were measured by co-staining cells with MitoTracker Green and Lysotracker Deep Red, followed by treatment with FCCP for 30 min before flow cytometry analysis. Additionally, autophagophore formation was evaluated by examining LC3 integrated density associated with mitochondria in cells transduced with LC3-mCherry and mito-GFP. Mitochondrial mass was determined by staining cells with MitoTracker Green. Furthermore, the Oxygen Consumption Rate (OCR) and the increase in Extracellular Acidification Rate (ECAR) were determined using Seahorse Real-Time Cell Metabolic Analysis. The experimental protocols for these assays were established in the laboratory, as previously described.^73^

ATP levels were quantified using the Colorimetric ATP Assay Kit (Abcam), following the manufacturer’s protocol, as previously described by us previously.^72^ The measurement was conducted on protein extracts obtained from CAR T cells and expressed as ATP content in nanograms per microgram of total T cell protein.

#### Calcium imaging

Fluorescently encoded Ca^2+^ sensors were used to measure the Ca^2+^ in cytosol or mitochondria. For Cytosolic Ca^2+^ measurement, cytosolic CGAMP6f (Addgene plasmid #163045) was used. For mitochondrial Ca^2+^ measurement, we used previously developed mitochondrial targeted Ca2+ sensor mito-RCaMP1h (Addgene, plasmid #105013). CD8^+^ T-cells obtained from control or TR mice were stimulated with Histamine (50μM) immediately within 30 s of live image recording and continued for 300 s. Images were collected at 3 s intervals using the 60x/1.42 oil immersion objective microscope with 488nm laser for CMV-Mito4x-GCaMP6f, and 561 nm laser for mito-RCaMP1h (Nikon Ti2E confocal microscope).

#### Immunoprecipitation of mTOR and activity assay

The immunoprecipitation of mTOR was carried out in isolated CD8^+^ CAR T cells following our previously established protocol.^72^ Total protein lysate was obtained from CD8^+^ CAR T cells and used for immunoprecipitation. In brief, 50μg of the entire lysate was mixed with 5μg each of mTOR antibody (Thermo Fischer Scientific) and incubated for 12 h at 4°C with continuous rotation at 360° on a shaker. Following this, Protein A/G Sepharose beads (Abcam), pre-blocked with 0.1% BSA, were added to the lysate in a 2:3 ratio and incubated for 1 h at room temperature. The purified protein was then utilized for the mTOR activity assay using the K-LISA mTOR Activity Kit (Merck Millipore) as per the provided instructions. Absorbance was measured at 450 nm using the VICTOR Nivo Multimode Microplate Reader (PerkinElmer), and the data is presented as the percentage of mTOR activity.

#### RNA sequencing and single cell analysis

RNA Sequencing and single-cell analysis procedures were conducted as follows: To prepare mRNA for bulk sequencing, CAR T cells undergoing various treatments were first enriched for mRNA using QIAGEN Pure mRNA beads. Following further enrichment and heat fragmentation, RNA was reverse-transcribed into cDNA, employing RNase H-Reverse Transcriptase and primers for strand-specific ligation. Adapters were then added to inserts in a strand-specific manner. libraries were then purified with QIAseq CleanStart PCR reagents for Illumina NovaSeqTM 6000 compatibility. Bulk RNA-seq data obtained in FastQ format underwent pre-processing and processing steps using nf-core pipeline. Differential gene expression (DEG) analysis between groups was done using iGeak_RNA-seq software. Functional Annotation, Pathway analysis and visualization (heatmaps, etc.) utilized online servers like DAVID, KEGG, ShinyGO and SR plot (Tables S2 and S3).

Additionally, publicly available scRNA-seq data for patient CAR T cells (ID: GSE168940) was analyzed in R, utilizing the Seurat package for data integration and normalization. Cell populations were automatically annotated using the SingleR package with the Human Primary Cell Atlas as reference database. UMAP dimensional reduction techniques were applied to visualize cellular heterogeneity and the distribution of autophagy-related gene expression across the identified cell clusters, with plots generated using the ggplot2 package (Tables S4 and S5).

#### Molecular docking

The X-ray crystal structure of human Atg4b (PDB ID: 2CY7) was obtained from the Protein DataBank (https://www.rcsb.org/) at a resolution of 1.90 Å. Following structural refinement, the active site was identified using the Computed Atlas of Surface Topography of Proteins (CASTp), which mapped key residues in the active pocket based on surface topology. These residues were further analyzed with the frustratometeR tool in R to identify highly frustrated regions linked to biologically significant activity. Multiple sequence alignment (MSA) was performed using ClustalW to ensure the selected residues are conserved through evolution. Three-dimensional structure of urolithin A molecule was retrieved from PubChem database with CID number 5488186. Molecular interactions were explored using PyrX, with AutoDock Vina as the scoring algorithm.

#### *In vivo* xenograft models and tumor re-challenge

On day 0, the NSG mice were injected with 0.5 x 10^6^ Raji cells via the tail vein. For the TR, repeated injections of Raji cells (0.2 x 10^6^) were given on day 12, day 19 and day 26 after the first injection. Post day 5 of the first Raji cells injection, the animals received an intravenous injection of 5 x 10^6^ CAR T cells. 24 mice were used in each group and blood samples were collected at various time points post-TR, specifically on days-14, 21, and 28, and human (h)CD3 cells were isolated using magnetic cell sorting (Miltenyi Biotech). Blood samples were pooled (from 3 mice) to isolate to hCD3 cells for the analysis of T cell markers, and functional and exhaustion markers such as Granzyme B, Perforin, PD-1, LAG-3. Further, measurement of IL-2 and IFN-γ levels was done via ELISA assay after co-culture of the isolated hCD3 cells with Raji cell *in vitro* for 24 h. Similarly, CD8^+^ T cells were isolated from pooled blood samples using magnetic selection (Miltenyi Biotec) and further analyzed by flow cytometry for marker analysis, including Granzyme B, Perforin, PD-1, LAG-3, CAR expression, and various T cell subsets such as T cell subsets as; T naive (Tn; CD45RO^−^ CCR7^+^), T stem cell memory (Tscm; CD45RO^−^ CCR7^+^), T central memory (Tcm; CD45RO^+^ CCR7^+^), T effector memory (Tem; CD45RO^+^ CCR7^-^) and T effector (Teff; CD45RO^−^ CCR7^-^). Additionally, the sorted CD8^+^ T-cells were subjected to FACS sorting (CD45RO^+^ expressing CAR^+^) and used for various analyses including Ca^2+^ imaging, mitochondrial metabolic measurements, mtDNA analysis, rate of mitophagy, autophagosome formation, and autophagy marker analysis. Furthermore, CAR T cells were evaluated by flow cytometry, and CAR DNA copy number was assessed via RT-qPCR using a Lenti-XTM Provirus Quantitation Kit (Takara).

Two different tumor models were developed using either Raji or NALM6 cells. On day 0, 1 x 10^6^ Raji cells or 1 x 10^6^ NALM6 were administered followed by the administration of 1 x 10^7^ (high dose) or 5 x 10^6^ (low dose) CAR T-cells on day 5 to Raji group. Similarly, for NALM6 model, a lower dose of CAR T-cells was used 5 x 10^6^. The CAR T-cells were cultured in the presence of 5 nM of SG and 10 μM of UrA for 7-day individual or in combination with fresh supplementation every 2-day nGLP-1 secreting CAR T (scGLP-1-CAR T) cells were also administered after being cultured with 10 μM UrA for 7-day *in vitro* before administration. Additionally, mice were treated with either vehicle (DMSO) or 10 mg/day of UrA same day post CAR T cell administration and continued throughout the experiment. Notably, we also directly administered 10 mg/day of UrA/day or a weekly injection of SG same day post CAR T cell injection and continued throughout the experiment, to assess their impact on CAR T-cells *in vivo*. Tumor burden was monitored using Bioluminescence imaging (BLI) with the IVIS Lumina LT *In Vivo* Imaging System (PerkinElmer). Anesthetized mice were injected with IVISbrite Bioluminescent Substrates (PerkinElmer) and imaged for 1 to 5 m, with bioluminescence intensity analysis conducted using the LT *In Vivo* Imaging System software. To assess the long-term persistence of CAR T cells, blood was collected weekly, and CAR T cell analysis was performed by flow cytometry and CAR copy number analysis using RT-qPCR (Lenti-X Provirus Quantitation Kit).

#### CRS mice model

The Cytokine Release Syndrome (CRS) mouse model was established using 4–6-week-old female mice sourced from Taconic. The strain, C.B.Igh-1b/GbmsTac-PrkdcscidLystbgN7 (SCID-beige), has been previously validated as an effective model for studying CRS. Mice were intraperitoneally injected with 1 x10^6^ Raji cells, and tumors were allowed to develop over 20-day. On day 21, 1 x10^7^ CAR T vehicle treated or nGLP-1 secreting and UrA treated CAR T (MCAR T-1) cells were administered via intraperitoneal injection, and blood samples were collected on day 24 for cytokine analysis.

#### Assessment of toxicity parameters

Toxicity parameters were assessed by collecting and processing blood to extract serum. The levels of Alkaline Phosphatase (ALP), Alanine transaminase (ALT), and Aspartate transaminase (AST) were determined in the blood samples from mice subjected to different CAR T cell treatments. These measurements were conducted two weeks after administering the Raji cell treatment.

### Quantification and statistical analysis

Statistical analysis was carried out using Prism version 8.0 (GraphPad Software). A non-parametric t-test was applied to assess differences among the various groups. Multiple group comparison, a non-parametric one-way ANOVA was used, and the Mantel-Cox test was done to compares survival between two groups. Prism software was utilized to generate bar graphs, including the corresponding *p* values. The data is expressed as the mean ± standard error of the mean (SEM). Statistical significance is represented as follows: ∗∗∗∗*p* < 0.001, ∗∗∗*p* < 0.005, ∗∗*p* < 0.01, and ∗*p* < 0.05. Unless stated otherwise in the figure legends, the study included at least three biological replicates.

## Supplemental information

Document S1. Figures S1–S23

Table S1. Excel file containing the autophagy and mitophagy reporter assay for the drug molecules, related to Figures 3A, 3B, and 3C

Table S2. Excel file containing the RNA-seq data, related to Figure 3G

Table S3. Excel file containing raw count data matrix from RNA-seq, related to Figure 3G

Table S4. Excel file containing the scRNA-seq data of autophagy-related gene expression across the identified cell clusters, related to Figures 2L and S10A

Table S5. Excel file containing the scRNA-seq data of all T cell populations, related to Figures 2L and S10A

Table S6. Excel file containing the oligonucleotide sequences, related to the key resource table

Document S2. Article plus supplemental information
