# Supplementary material for: Bioengineering the metabolic network of CAR T cells with GLP-1 and Urolithin A increases persistence and long-term anti-tumor activity
Source: Cell Rep Med. 2025 Mar 18;6(3):102021. doi: 10.1016/j.xcrm.2025.102021 (PMC11970383; doi:10.1016/j.xcrm.2025.102021)
Supplement: Document S1. Figures S1–S23 [file mmc1.pdf]

Cell Reports Medicine, Volume *6*

Supplemental information

Bioengineering the metabolic network of CAR T cells with GLP-1 and Urolithin A increases persistence and long-term anti-tumor activity

Areej Akhtar, Md Shakir, Mohammad Sufyan Ansari, Divya, Md Imam Faizan, Varnit Chauhan, Aashi Singh, Ruquaiya Alam, Iqbal Azmi, Sheetal Sharma, Mehak Pracha, Insha Mohi Uddin, Uzma Bashir, Syeda Najidah Shahni, Rituparna Chaudhuri, Sarah Albogami, Rik Ganguly, Shakti Sagar, Vijay Pal Singh, Gaurav Kharya, Amit Kumar Srivastava, Ulaganathan Mabalirajan, Soumya Sinha Roy, Irfan Rahman, and Tanveer Ahmad

Supplementary Figures:

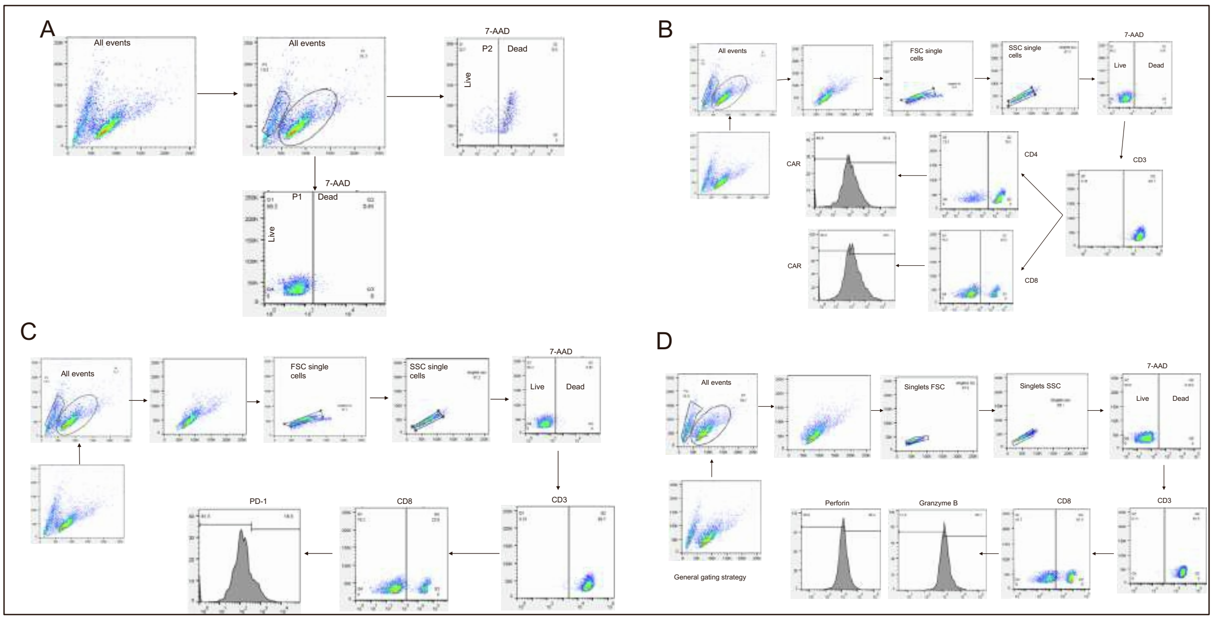

Figure S1: Flow cytometric analysis of CAR T cell populations and activation markers performed using a sequential gating strategy. Initially, lymphocytes were identified based on FSC-A and SSC-A. Doublet discrimination was done by plotting FSC-H against FSC-A, followed by SSC-H versus SSC-A. Dead cells were excluded by gating on 7-AAD-negative populations (A). Live, CD3-positive cells were then selected and further gated into $CD4^+$ and $CD8^+$ subpopulations followed by CAR detection in these populations (B). Similarly, gating strategy to identify the PD-1 (C) and Granzyme B and perforin (D). This gating strategy ensured the accurate identification of live/dead cells and T cell marker analysis allowing for a detailed characterization of their function and exhaustion states.

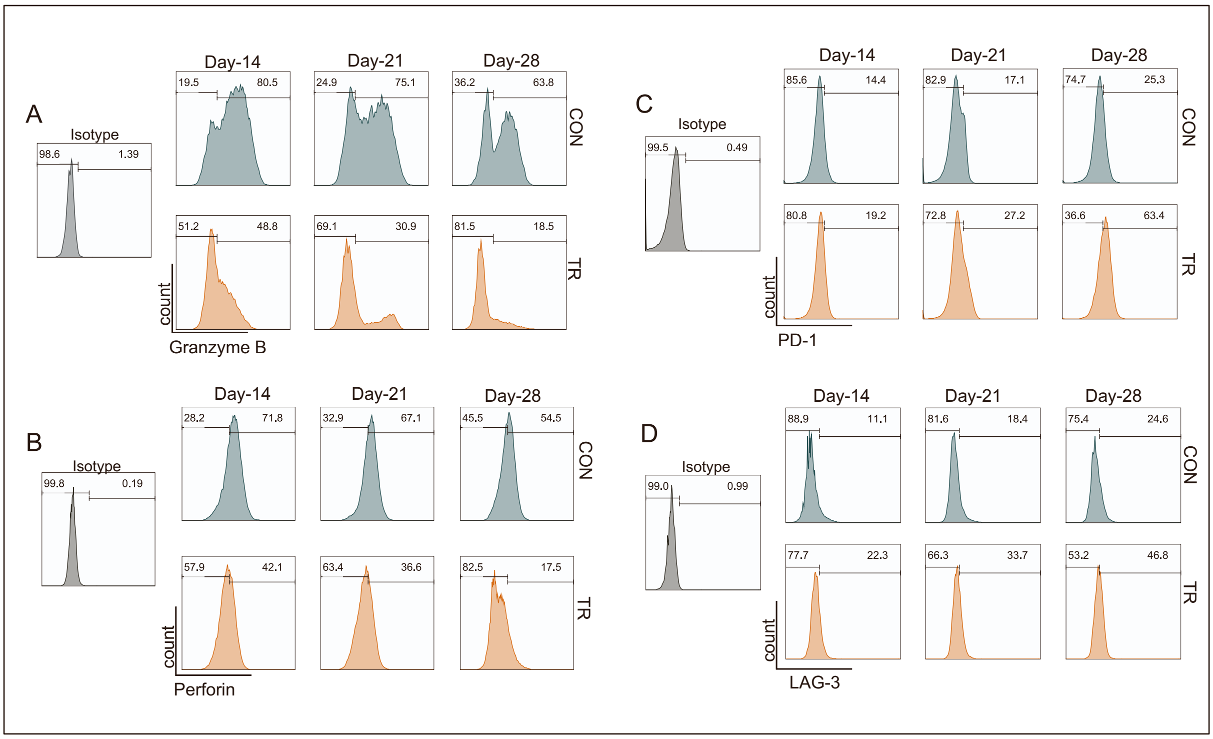

Figure S2: CAR T cell dysfunction triggered upon tumor re-challenge (TR)

(A-B) Flow cytometric analysis showing histogram plots of Granzyme B and Perforin in CAR T cells from CON and TR groups at days 14, 21, and 28. The y-axis represents the cell count and x-axis represents the anti-granzyme and anti-perforin antibody fluorescence signal represented as Granzyme B or Perforin.

(C-D) Histogram plots of PD-1 and LAG-3 in CAR T cells from CON and TR groups at days 14, 21, and 28.

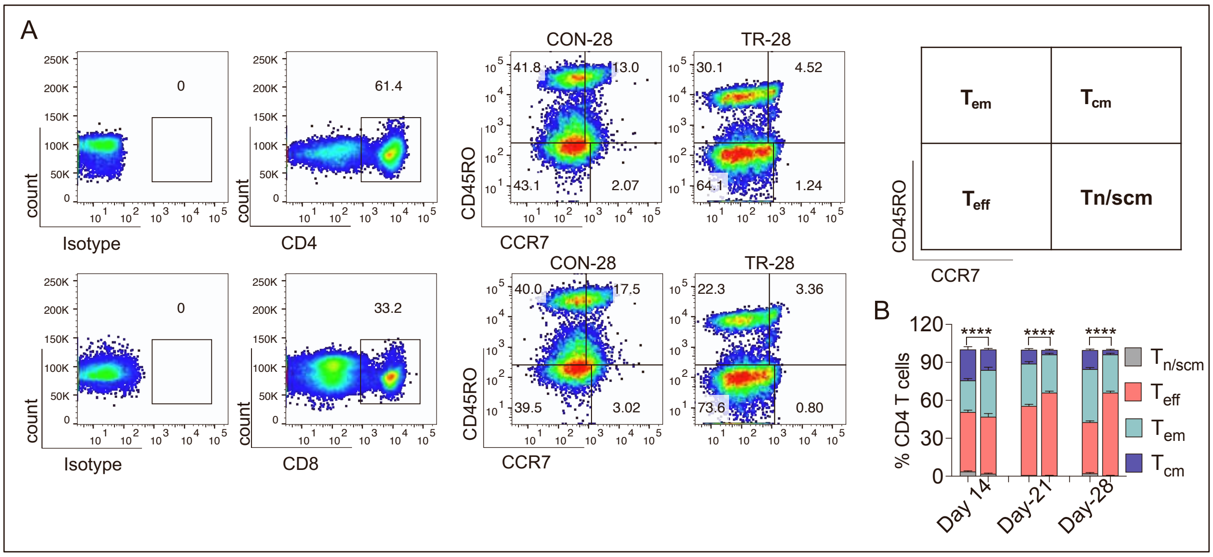

Figure S3: CAR T cell immunophenotyping upon tumor re-challenge (TR)

(A) Flow cytometric analysis of CAR T cells for various markers after TR for 21 days. The cells were first stained for CD4 or CD8 and then for various T cell subsets using the antibodies against CCR7 and CD45RO.

(B) Histogram analysis displaying of various CD4 T cell subsets.

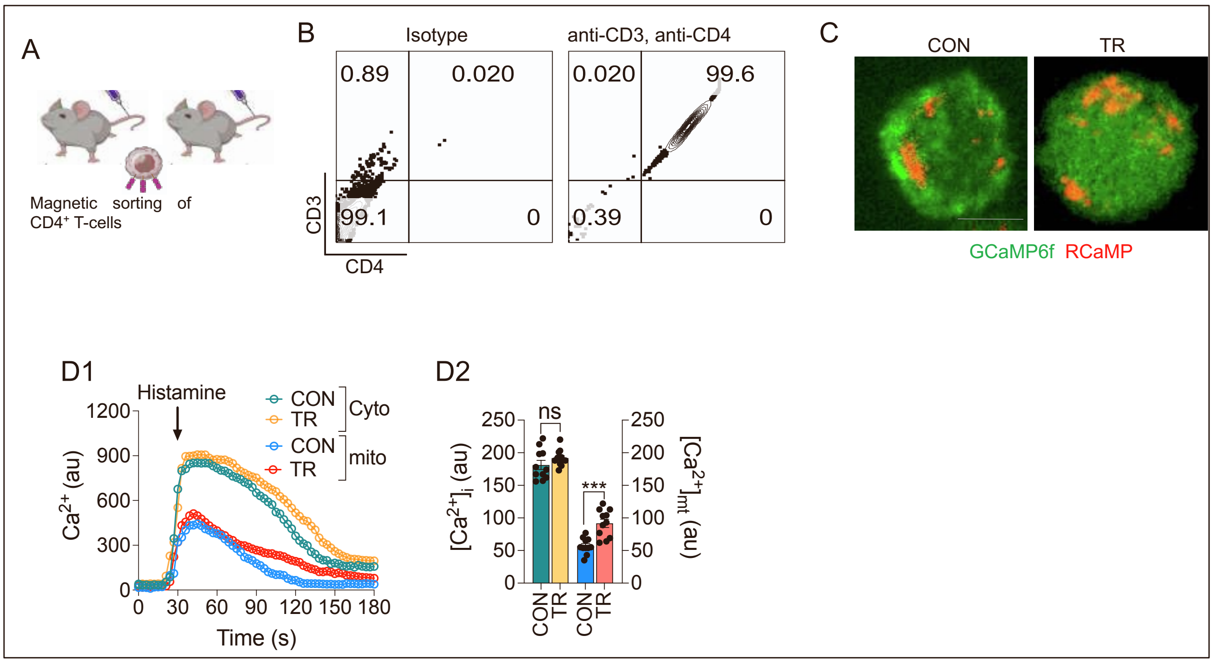

Figure S4: Measurement of intracellular calcium $[Ca^{2+}]_i$ and mitochondrial calcium $[Ca^{2+}]_{mt}$ in CD4$^+$ T cells obtained from CON and TR mice model

(A) CD4$^+$ T cells were isolated from pooled blood samples of tumor-bearing mice using magnetic sorting.

(B) Representative flow cytometric plots showing the purity of CD4$^+$ T cell populations after magnetic sorting.

(C) Representative images from live cell imaging of CAR T cells showing mitochondrial calcium $[Ca^{2+}]_{mt}$ (RCaMP, red) and cytosolic calcium $[Ca^{2+}]_i$ (GCaMP6f, green) in CD4$^+$ T cells of CON and TR mice.

(D1) Real-time changes in cytosolic $[Ca^{2+}]_i$ and mitochondrial $[Ca^{2+}]_{mt}$ levels in control (CON) and tumor re-challenged (TR) CD4$^+$ T cells following histamine stimulation.

(D2) Bar graphs summarizing the average $[Ca^{2+}]_i$ and $[Ca^{2+}]_{mt}$ levels in CD4$^+$ T cells from CON and TR mice (n=12 data points).

Data represents mean ± SE; from three independent experiments.***$p < 0.001$; ns (not significant). A non-parametric t-test was used for statistical analysis. Scale bar: 10 µm.

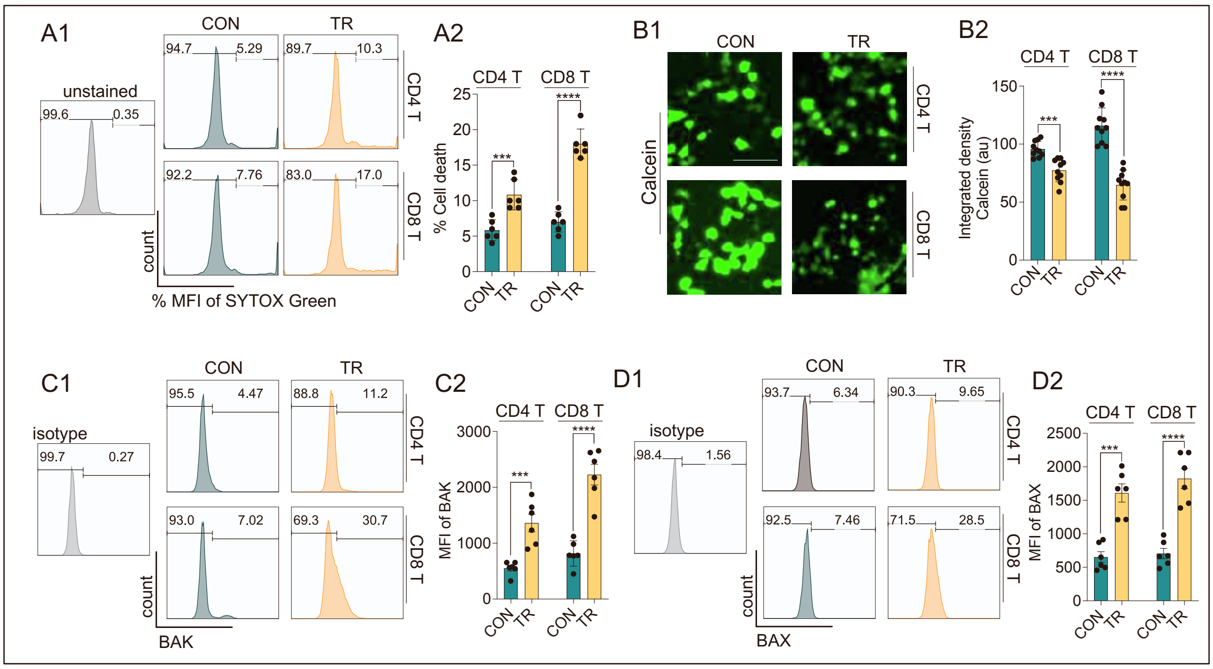

Figure S5: Analysis of cell death and mitochondrial apoptotic markers in T cells from tumor re-challenged and control mice

(A1-A2) Flow cytometric analysis of SYTOX Green staining in $CD4^+$ and $CD8^+$ T cells from control (CON) and tumor re-challenged (TR) groups (n=6).

(B1-B2) Fluorescence microscopy images of calcein-AM stained $CD4^+$ and $CD8^+$ T cells from CON and TR groups (n=10 images).

(C1-C2) Flow cytometric analysis of mitochondrial BAK expression in $CD4^+$ and $CD8^+$ T cells from CON and TR groups (n=6).

(D1-D2) Flow cytometric analysis of BAX expression in $CD4^+$ and $CD8^+$ T cells from CON and TR groups (n=6).

Data represents mean ± SE; from three independent experiments. ***$p < 0.001$; ****$p < 0.0001$. A non-parametric t-test was used for statistical analysis. Scale bar: 50 µm.

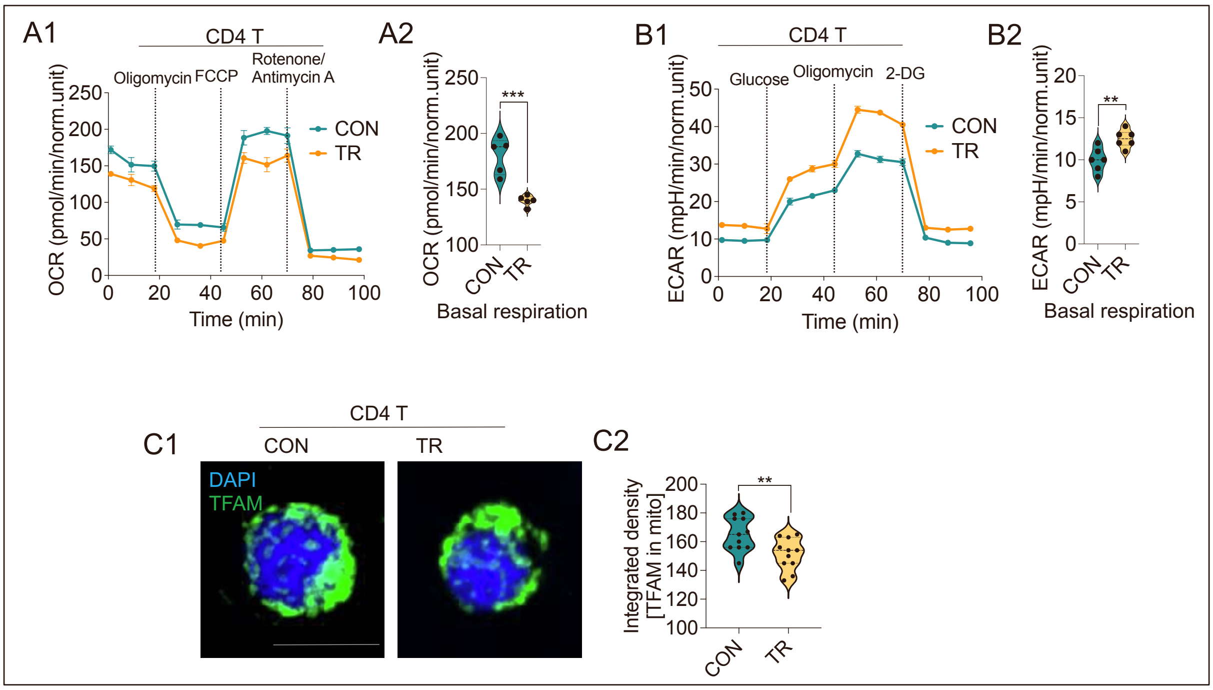

Figure S6: Metabolic and mitochondrial DNA assessment of CD4⁺ T cells from CON and TR mice

(A1) Seahorse assay measuring the Oxygen Consumption Rate (OCR) in CD4⁺ T cells from CON and TR groups over time in response to the treatments. (A2) Bar graph shows the basal respiration (n=5).

(B1) Seahorse assay measuring Extracellular Acidification Rate (ECAR) in CD4⁺ T cells from CON and TR groups. Increased ECAR levels in TR cells indicate a metabolic shift towards glycolysis, compensating for the reduced oxidative phosphorylation post-TR. (B2) Bar graph shows the basal respiration (n=5).

(C1-C2) TFAM expression: (C1) Representative immunofluorescence images of CD4⁺ T cells stained for TFAM (mitochondrial transcription factor) and DAPI showing a decrease in mitochondrial TFAM expression in TR cells compared to CON cells. (C2) Quantification of TFAM intensity (n=11).

Data represents mean ± SE; from three independent experiments. $^{**}p < 0.01$. A non-parametric t-test was used for statistical analysis. Scale bar: 10 μm.

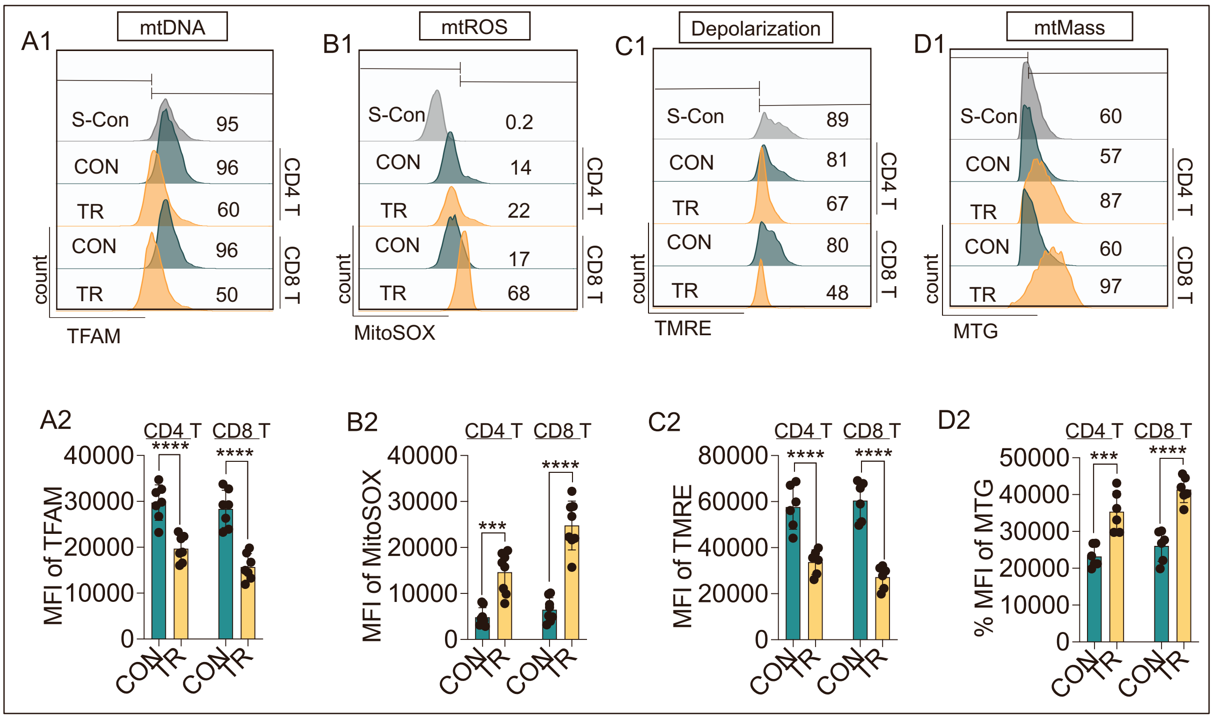

Figure S7: Mitochondrial mass, mtDNA, membrane potential (ΔΨm), and ROS levels in CD4⁺ and CD8⁺ T cells from CON and TR mice

(A1-A2) Histogram and MFI analysis of TFAM expression as a marker for mtDNA content in $CD4^+$ and $CD8^+$ T cells (n=7).

(B1-B2) Histogram and MFI analysis using MitoSOX to detect mtROS levels in $CD4^+$ and $CD8^+$ T cells (n=7).

(C1-C2) TMRE staining was used to assess mitochondrial membrane potential. TR cells exhibited a marked reduction in ΔΨm, indicating mitochondrial depolarization compared to CON cells (n=6).

(D1-D2) MitoTracker Green (MTG) staining was performed to assess mitochondrial mass (n=6).

Data represents mean ± SE; from three independent experiments. ***$p < 0.001$; ****$p < 0.0001$. A non-parametric t-test was used for statistical analysis.

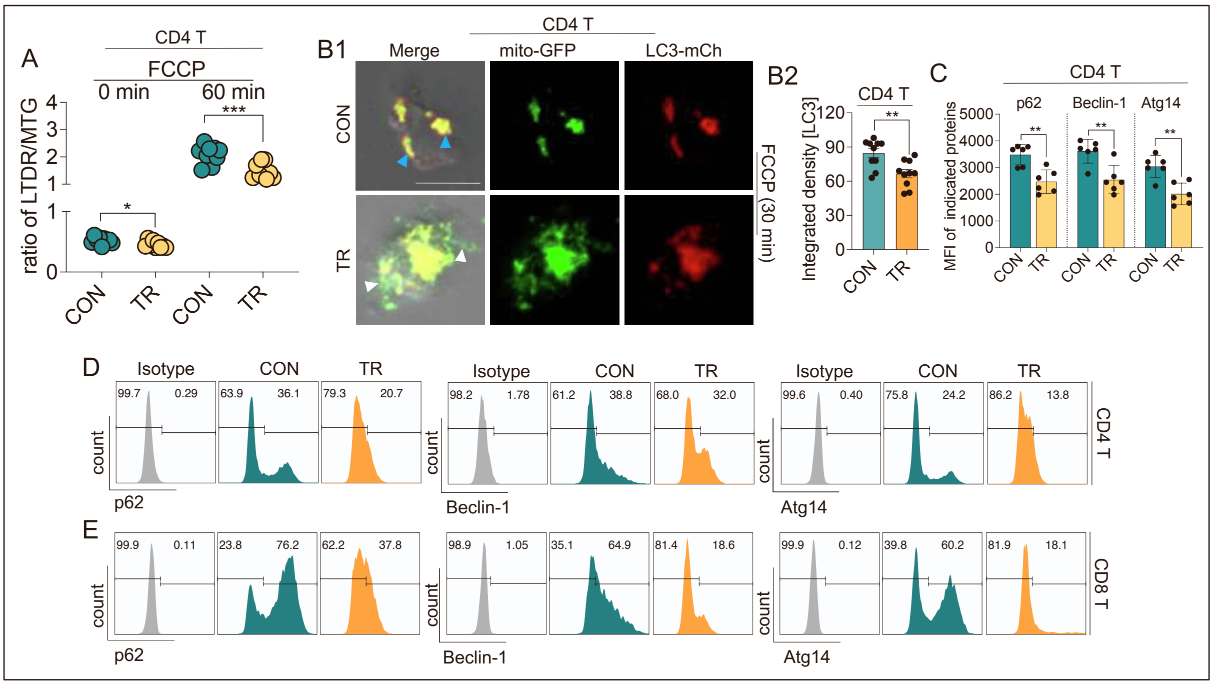

Figure S8: Mitochondrial dysfunction and autophagy-related impairments in CON and TR mice

(A) The ratio of LTDR to MTG fluorescence used to assess mitophagy in $CD4^+$ T cells from CON and TR mice after a 60 min FCCP treatment.

(B1-B2) Representative images of autophagosomes in $CD4^+$ T cells using LC3-mCherry and mito-GFP staining. Co-localization of mito-GFP with LC3-mCherry was significantly reduced in TR cells, indicating impaired autophagosome formation. FCCP treatment was given for 30 min.

(C) MFI of autophagy markers in $CD4^+$ T cells (n=6).

(D-E) Flow cytometry analysis of p62, Beclin-1, and Atg14 in CD4+ and $CD8^+$ T cells in TR cells compared to CON cells.

Data represents mean ± SE; from three independent experiments. $*p < 0.05$; $**p < 0.01$; $***p < 0.001$. A non-parametric t-test was used for statistical analysis. Scale bar: 10 µm.

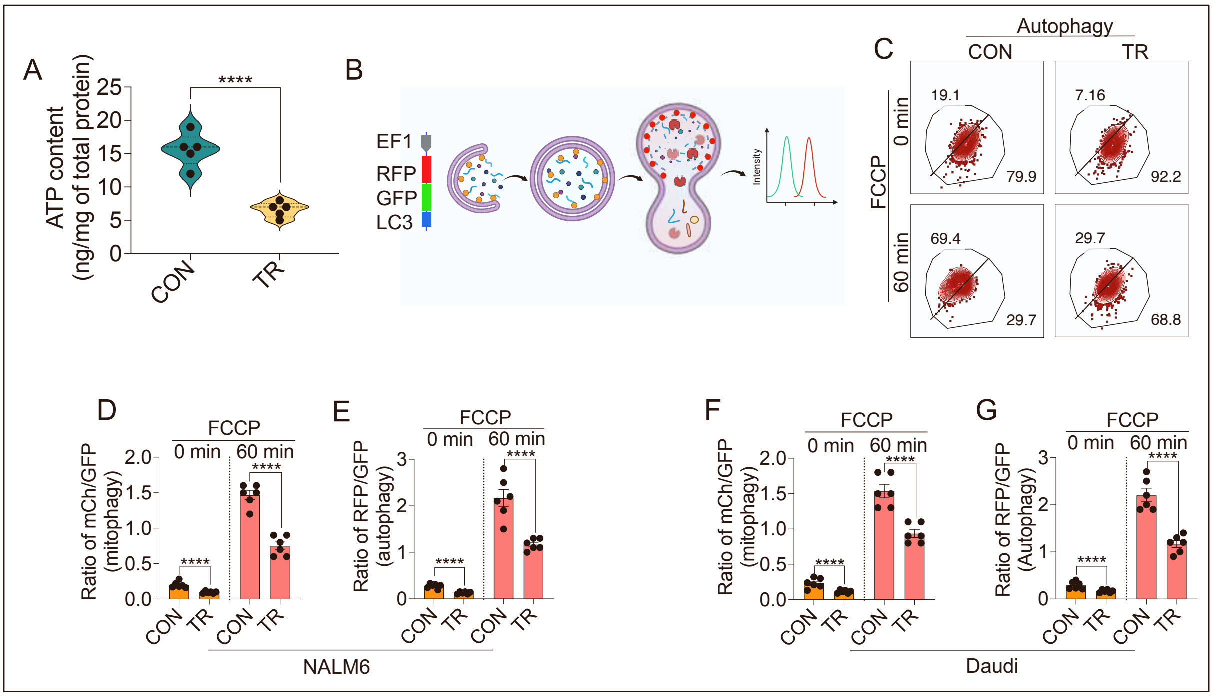

Figure S9: Mitophagy and autophagy impairments in CD8⁺ CAR T cells exposed to TR in vitro

(A) ATP content of CD8+ CAR T cells - TR compared to CON cells.

(B) Schematic of reporter assay used to evaluate autophagy. The reporter measures the change in fluorescence intensity, where the decrease in GFP signal (green) relative to the RFP signal (red) indicates autophagy.

(C) Flow cytometry analysis of the autophagy reporter in TR cells compared to CON cells, measured at both 0 and 60 minutes post-FCCP treatment.

(D-E) The mitophagy and autophagy reporter assay results for $CD8^+$ CAR T cells stimulated by NALM6 leukemia cells in TR cells compared to CON cells (n=9).

(F-G) The mitophagy and autophagy reporter assay results for $CD8^+$ CAR T cells stimulated by Daudi Burkitt's lymphoma cells in TR cells compared to CON cells (n=9).

Data represents mean ± SE; from three independent experiments. ****$p < 0.0001$. A non-parametric t-test was used for statistical analysis.

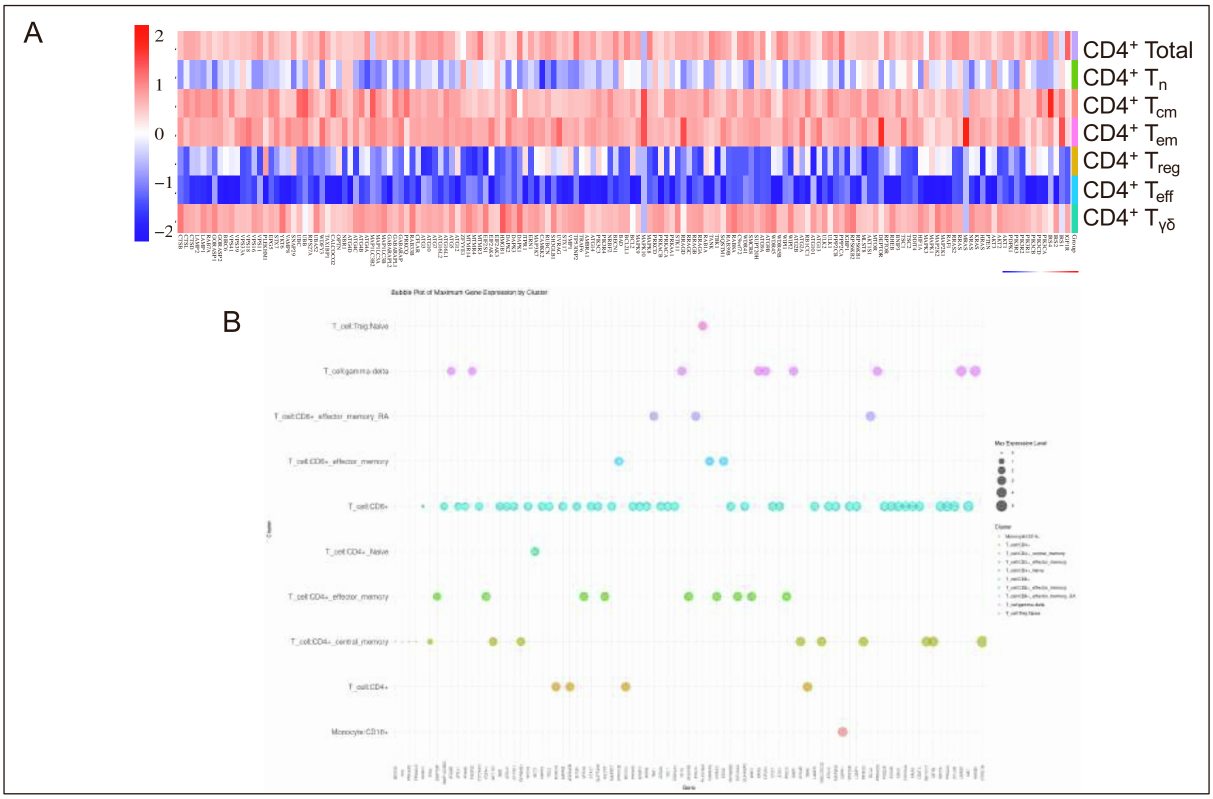

Figure S10: Gene expression across various T cell subsets derived from existing single-cell RNA seq data

(A) Heatmap showing the differential expression of autophagy-related genes across multiple T cell subsets, as assessed from existing single-cell RNA sequencing data.

(B) Bubble plot showing the maximum gene expression by cluster. Each dot represents a cluster, with size corresponding to the maximum expression level of autophagy genes.

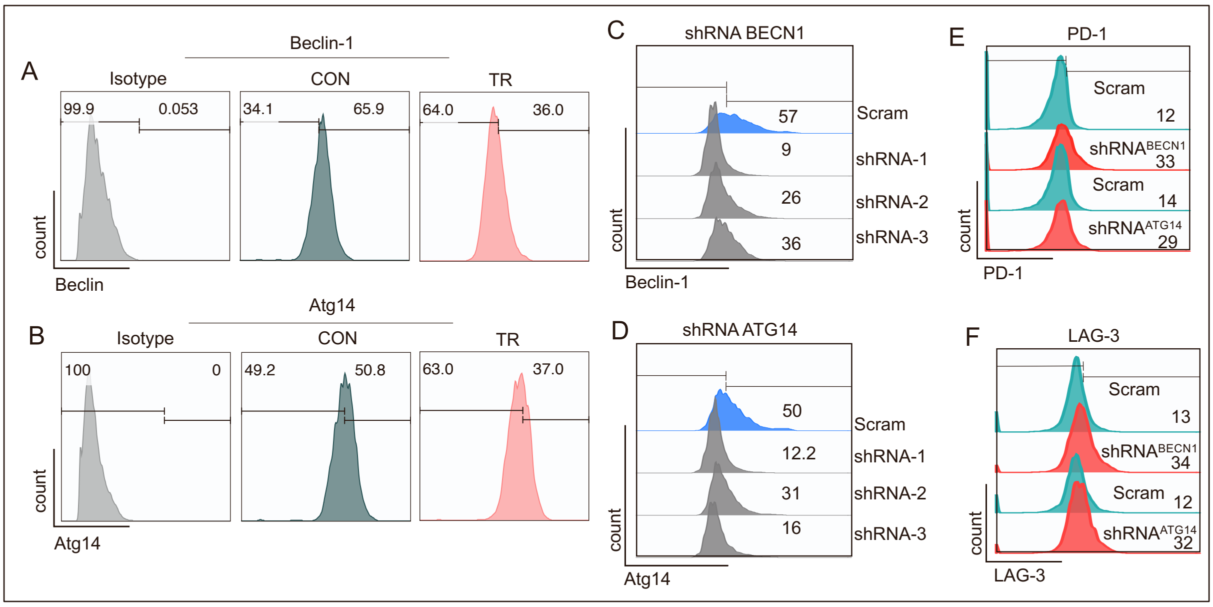

Figure S11: Downregulation of autophagy-related proteins ATG14 and Beclin-1 impairs CAR T cell function and induces exhaustion

(A-B) Representative histograms showing mean fluorescence intensity (MFI) of Atg14 and Beclin-1 expression in CAR T cells from CON and TR mice on day 21 post-TR.

(C-D) Validation of shRNA knockdown efficiency of Beclin-1 and Atg14 in CAR T cells at day 7 post-transduction. Three shRNA clones for each gene (shRNA-1, shRNA-2, and shRNA-3) were tested, and knockdown was confirmed by flow cytometry, showing significant reductions in Beclin-1 and Atg14 expression compared to scrambled control.

(E-F) Flow cytometry analysis of PD-1 and LAG-3 exhaustion markers in CAR T cells with Beclin-1 or Atg14 knockdown at day 21 post-TR. Knockdown of either protein led to a significant increase in exhaustion marker expression compared to the scrambled control, similar to cells under TR conditions.

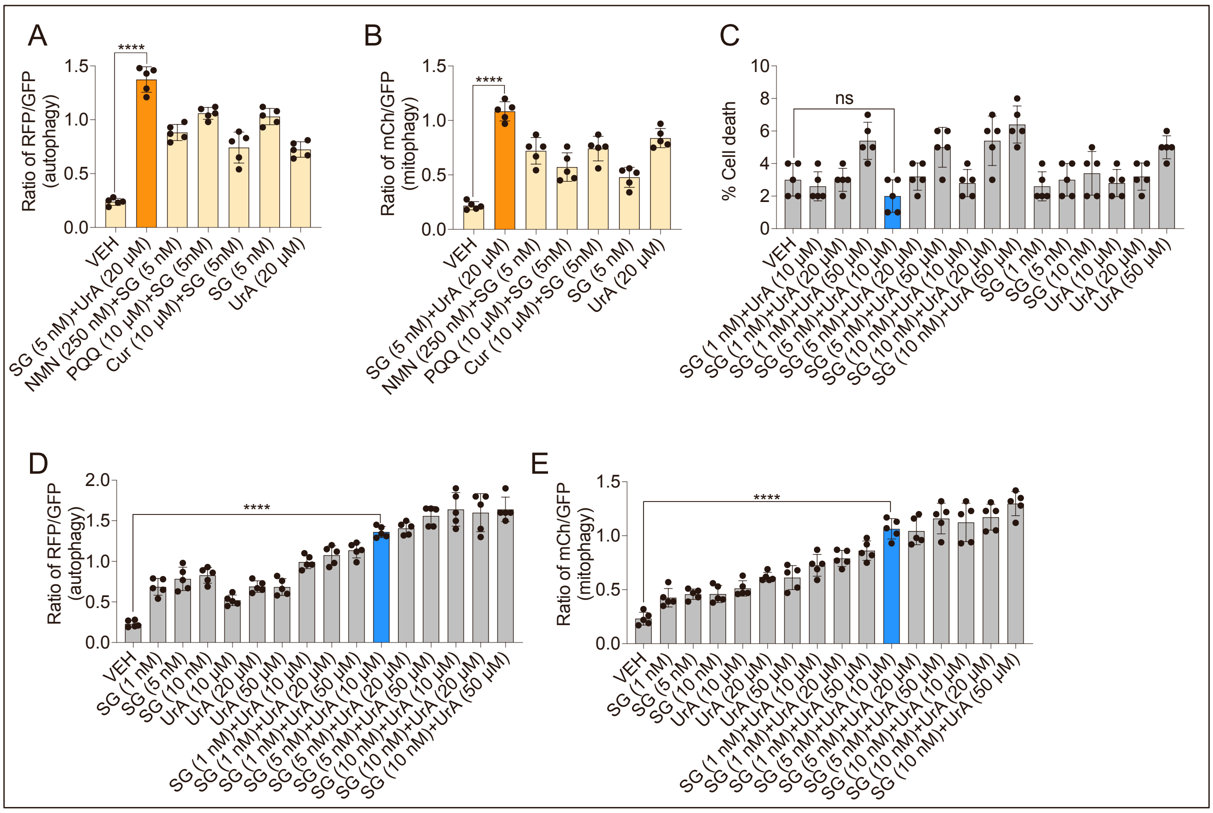

Figure S12: Induction of autophagy and mitophagy in CAR T cells by SG and UrA combination

(A-B) Flow cytometry-based analysis using the RFP/GFP ratio for autophagy (A) and the mCherry/GFP ratio for mitophagy (B) in CAR T cells treated with respective drugs/combinations (n = 5).

(C) Percentage of cell death as assessed by flow cytometry. Data are presented as mean ± SD of (n = 5).

(D-E) Dose titration analysis of SG (1-10 nM) and UrA (10-50 µM) in CAR T cells demonstrating the induction of autophagy (D) and mitophagy (E) (n = 5).

Data represents mean ± SE; from three independent experiments. ****$p < 0.0001$; ns (not significant). A non-parametric t-test was used for statistical analysis.

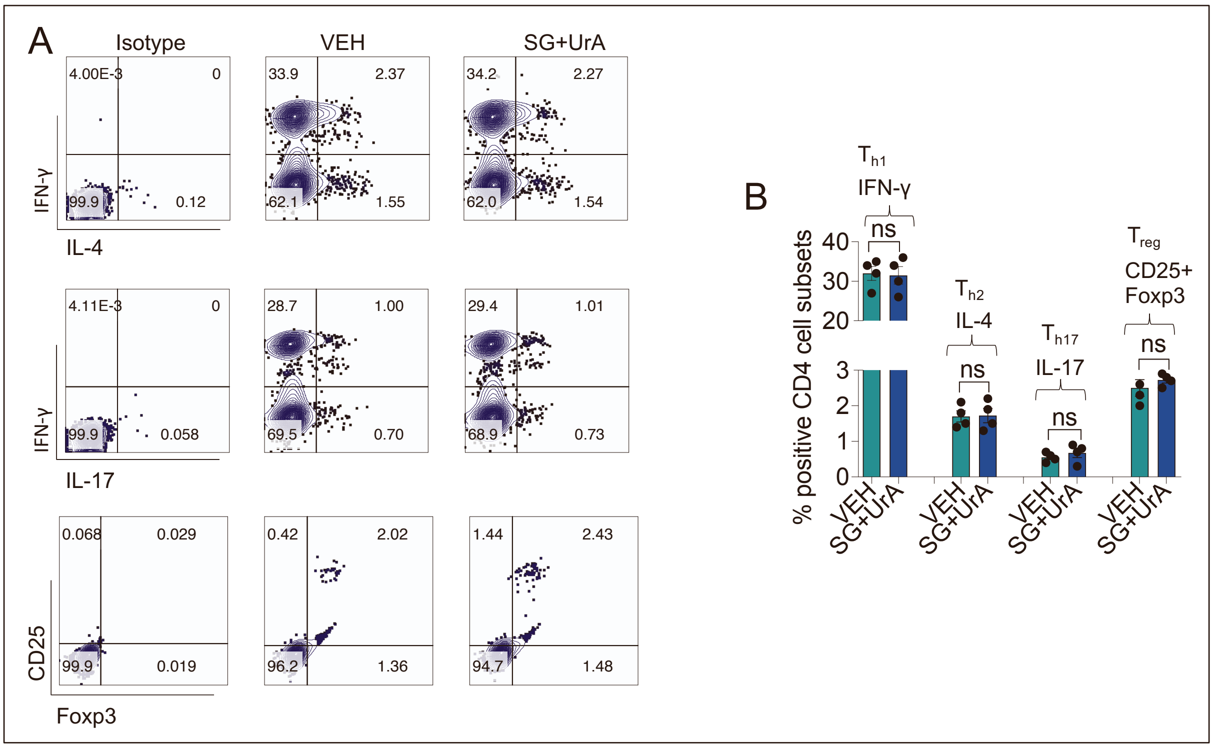

Figure S13: Evaluation of CD4 T cell polarity upon SG and UrA combination treatment

(A) Flow cytometry- analysis showing contour plots of various CD4$^+$ CAR T cell subsets upon induction with VEH and SG+UrA. IFN-γ, represents Th1 cells, IL-4 represents Th2 cells, IL-17 for Th17, and CD25+Foxp3 for Treg cells.

(B) Histogram analysis of the flow cytometry plots. Data are presented as mean ± SD of (n = 4). A non-parametric t-test was used for statistical analysis; ns (not significant).

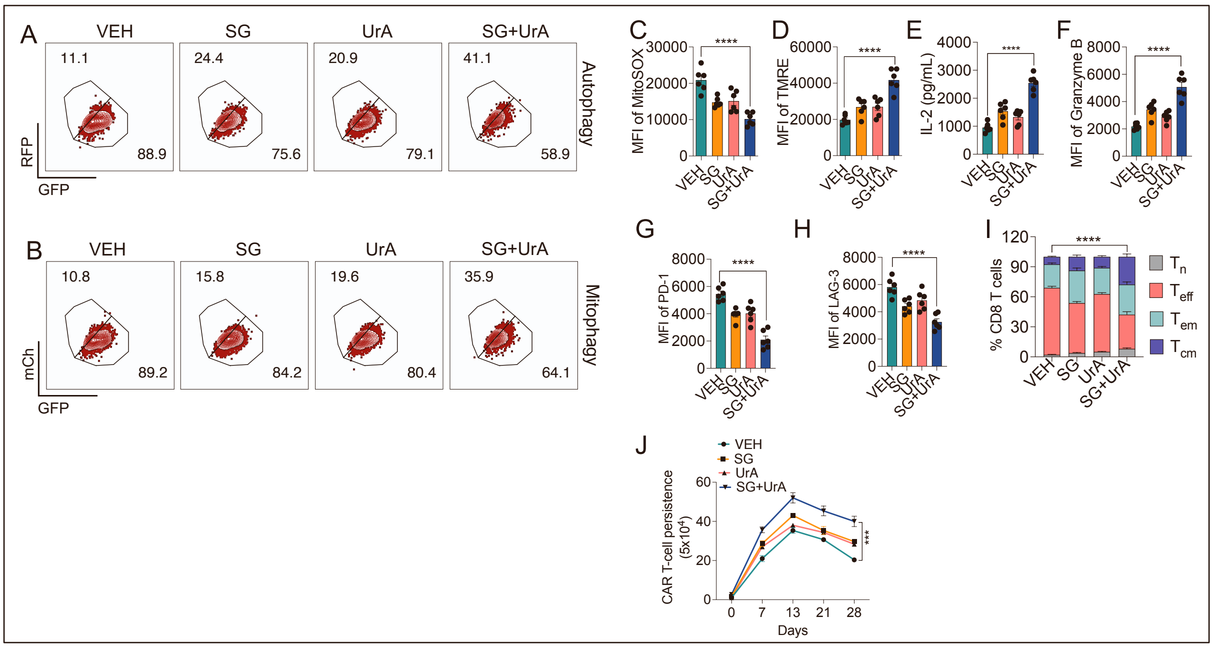

Figure S14: SG and UrA combination enhances autophagy, mitophagy, and mitochondrial function, while reducing exhaustion markers in CAR T cells.

(A-B) Flow cytometry analysis of autophagy (A) and mitophagy (B) in CAR Tcells cultured with SG (5 nM), UrA (10 µM) or their combination for 21 days. The percentage of autophagy is measured by RFP/GFP ratio (A), and mitophagy is measured by mCherry/GFP ratio (B). CAR T cells treated with SG+UrA showed significantly higher levels of both autophagy and mitophagy compared to VEH, SG, or UrA alone.

(C-D) Mitochondrial ROS (C) and mitochondrial membrane potential (D) measured by flow cytometry using MitoSOX and TMRE, respectively (n=6).

(E-F) Functional assays showing increased IL-2 secretion (E) and Granzyme B expression (F) in CAR T cells treated with SG+UrA compared to VEH, SG, or UrA alone (n=6).

(G-H) Flow cytometry analysis of exhaustion markers showing significant reductions in PD-1 (G) and LAG-3 (H) expression in CAR T cells treated with SG+UrA compared to VEH, SG, or UrA alone (n=6).

(I) T cell subset distribution (T_n, T_{eff}, T_{em}, T_{cm}) within the $CD8^+$ T cell population (n=6).

(J) CAR T cell persistence over 28 days. CAR T cells treated with SG+UrA demonstrated significantly higher persistence compared to VEH, SG, or UrA alone (n=6).

Data represents mean ± SE; from three independent experiments. ***$p < 0.001$; ****$p < 0.0001$. A non-parametric t-test was used for statistical analysis. Non-parametric one-way ANOVA was used to analyse the T cell subsets (panel I).

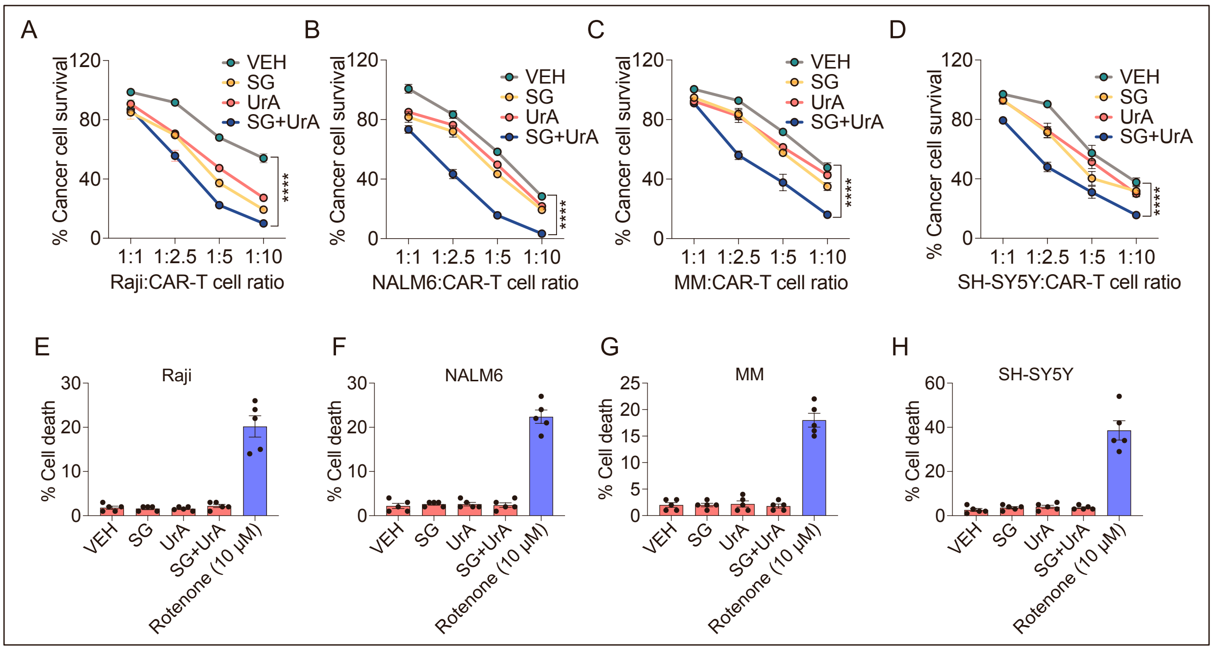

Figure S15: SG and UrA combination enhances anti-tumor activity in CAR T cells in multiple cancer models.

(A-D) Co-culture assays showing the percentage of cancer cell survival in co-culture with CAR T cells treated with VEH, SG (5 nM), UrA (10 µM), or the SG+UrA combination at different CAR T cell to target cell ratios. (A) Raji cells co-cultured with CD19 CAR T cells. (B) NALM6 cells co-cultured with CD19 CAR T cells. (C) BCMA-directed CAR T cells in co-culture with multiple myeloma (MM) cells. (D) GD2-directed CAR T cells against SH-SY5Y neuroblastoma cells. Across all models, the combination of SG+UrA showed a significantly higher reduction in cancer cell survival compared to single treatments or vehicle (n=6).

(E-H) Percentage of cell death in various tumor cell lines, including Raji (E), NALM6 (F), MM (G), and SH-SY5Y (H), treated with SG, UrA, SG+UrA, or Rotenone (10 µM) as a positive control (n=6).

Data represents mean ± SE; from three independent experiments. ****$p < 0.0001$. A non-parametric t-test was used for statistical analysis. The Mantel-Cox test was done to compares survival between two groups.

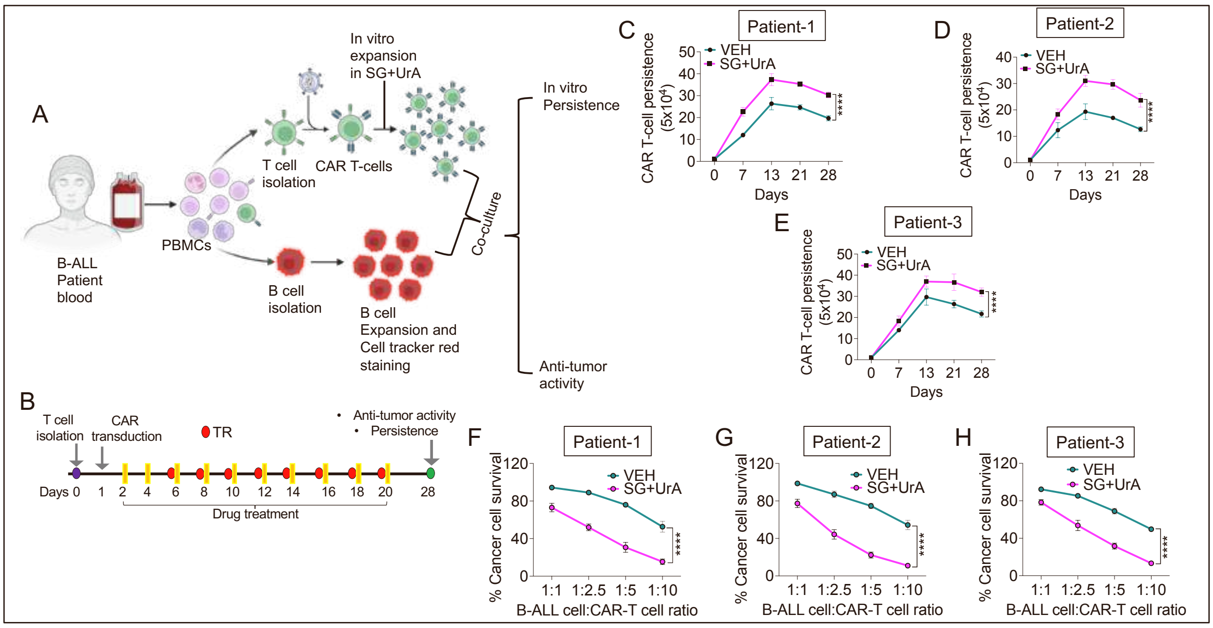

Figure S16: Isolation and functional assessment of patient-derived CAR T cells and Leukemic B-cells. (A) Illustration of the T and B cell isolation from the same patient blood samples. The T and B cells were separated using the magnetic isolation method.

(B) Illustration of TR model. Patient B-ALL cells were added every 2 days till 20 days and then the anti-tumor activity and CAR T cell persistence was evaluated on day 28.

(C-E) CAR T cell persistence assay in the co-culture of patient derived CAR T cells with the patient derived B cells for the indicated time points. The cells were obtained from three different patients diagnosed with B-ALL.

(F-H) CAR T cell anti-tumor activity in the co-culture patient derived CAR T cells with the patient derived B cells for the indicated time points.

Data represents mean ± SE; from three independent experiments. ****$p < 0.0001$. A non-parametric t-test was used for statistical analysis.

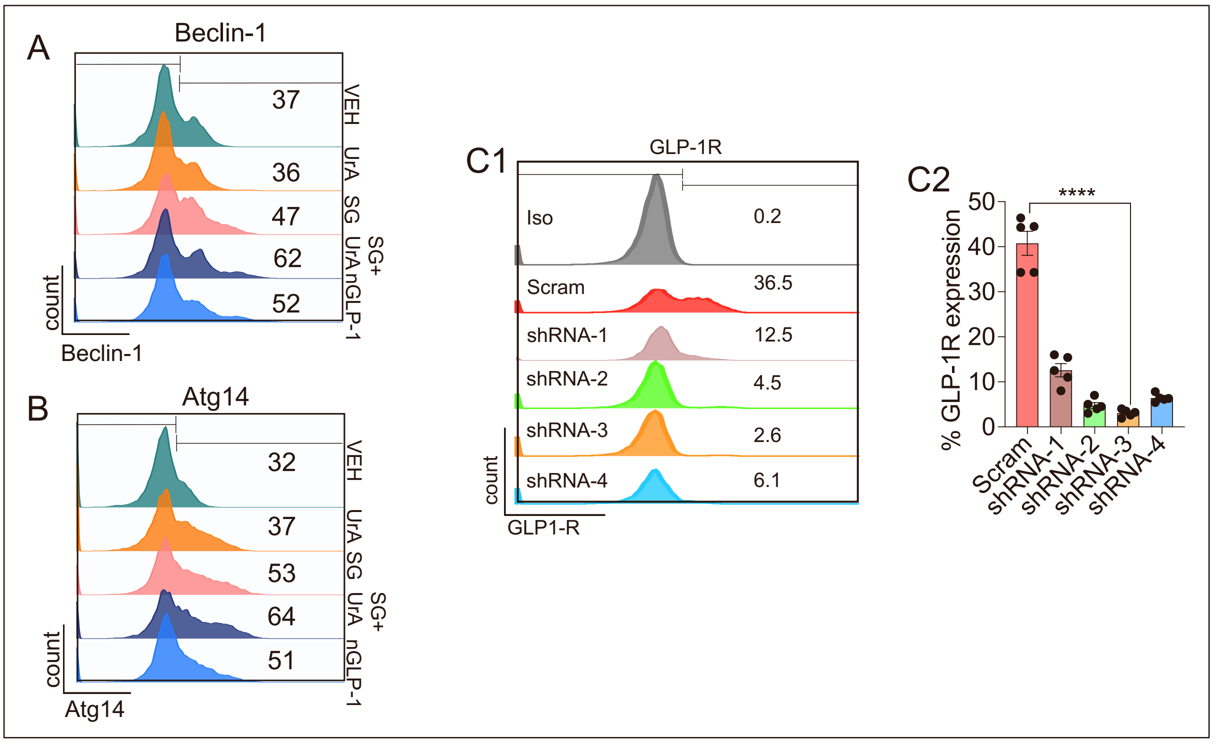

Figure S17: GLP-1R knockdown in T cells

(A) Histogram depiction of the expression of Beclin-1 in 21 day TR CAR T cells treated with VEH; UrA; SG; SG+UrA or nGLP-1. The bar graph of this representative histogram is shown in *Figure 3H.*

(B) Similarly, histogram depiction of the expression of Atg14. The bar graph of this representative histogram is shown in *Figure 3I.*

(C1-C2) Flow cytometric analysis showing the knockdown efficiency of GLP-1R in T cells. (C1) Histogram plots of GLP-1R expression in T cells transduced with scrambled control (Scram) or GLP-1R-targeting shRNA constructs (shRNA-1 to shRNA-4). (C2) Quantification of GLP-1R expression as a percentage of positive cells (n=5).

Data represents mean ± SE; from three independent experiments. ****$p < 0.0001$. A non-parametric t-test was used for statistical analysis.

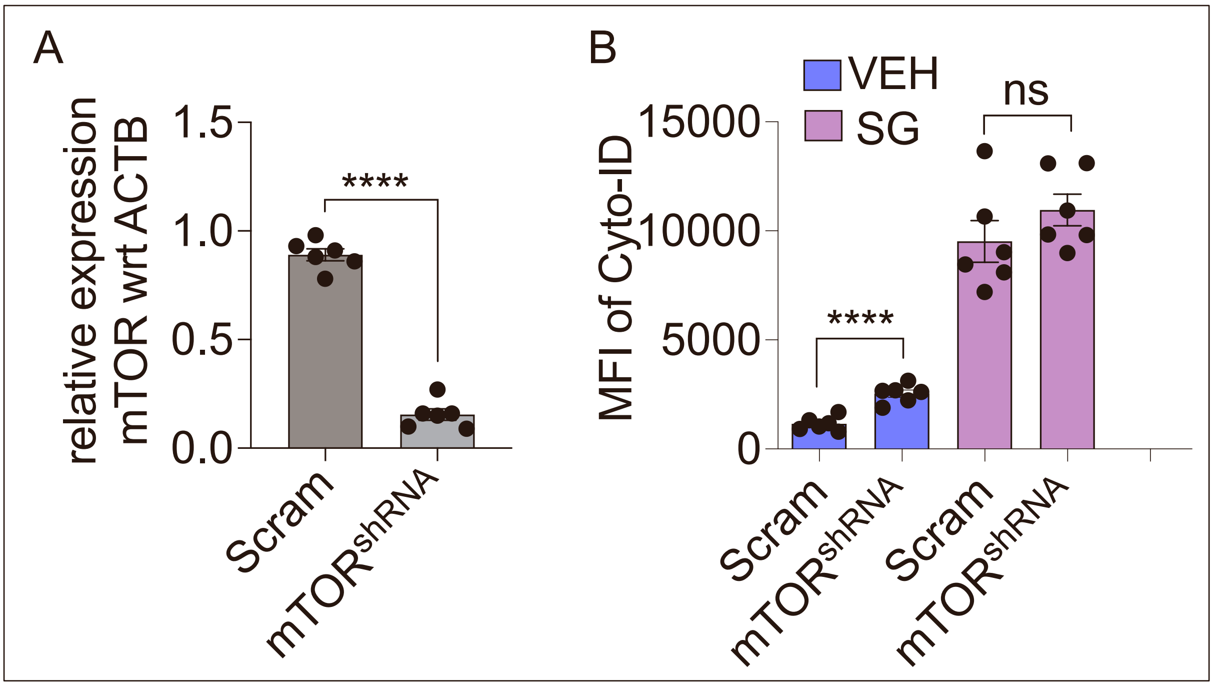

Figure S18: mTOR knockdown leads to increased autophagy, independent of GLP-1R activation.

(A) RT-qPCR analysis of mTOR expression with respect to the ACTB gene in CAR -cells transduced with Scram or mTOR-shRNA (n=6).

(B) Flow cytometry-based analysis of autophagy (% MFI) in cells transduced with mTOR-shRNA or scrambled control, and treated with either vehicle (VEH) or SG (n=6).

Data represents mean ± SE; from three independent experiments. ****p < 0.0001; ns (not significant). A non-parametric t-test was used for statistical analysis.

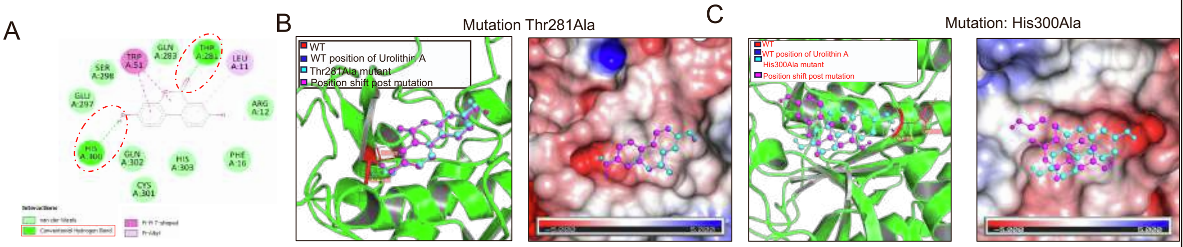

D

Sl No.	Position	WT	Mutant	ΔΔG	pH	T	RSA
1	281	T	A	-2.30	7.0	25	21.6
2	281	T	G	-2.63	7.0	25	21.6
3	300	H	A	-1.16	7.0	25	2.2
4	300	H	G	-0.82	7.0	25	2.2

E

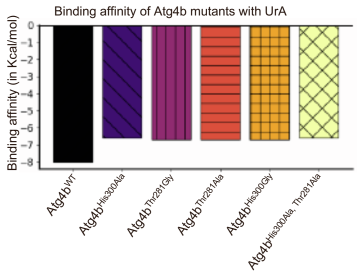

F

Sl. No	Receptor	Ligand	Binding Affinity (in Kcal/mol)
1.	Atg4b^{WT}	UrA	-8.0
2.	Atg4b^{Thr281Ala}	UrA	-6.6
3.	Atg4b^{Thr281Gly}	UrA	-6.7
4.	Atg4b^{His300Ala}	UrA	-6.7
5.	Atg4b^{His300Gly}	UrA	-6.7
6.	Atg4b$^{His300Ala, Thr281Ala}$	UrA	-6.6

Figure S19: Molecular interaction analysis between Urolithin A and ATG4B based on the docking studies. (A) The structure of ATG4B bound to UrA, highlighting interactions between the protein and the small molecule. Red dotted lines depict conventional hydrogen bonds, while other interactions include van der Waals forces and π-stacking.

(B) On the left, the superimposed structures of ATG4BWT and the ATG4BThr281Ala mutant are shown, both bound to UrA. On the right, UrA is displayed within the Coulombic charge surface of the active pocket in these superimposed proteins.

(C) The left panel shows the superimposed structures of ATG4BWT and the ATG4BThr281Gly mutant, again bound to UrA. The right panel depicts UrA within the Coulombic charge surface of the active pocket of these superimposed structures.

(D) Table summarizing alanine and glycine scanning mutagenesis results for amino acid positions 281 and 300 of ATG4B. It provides insights into how these mutations affect the protein's stability, focusing on changes in ΔΔG and the influence of temperature, pH, and Relative Solvent Accessible Area (RSA) on the protein's structure and energetics.

(E) A comparison of the binding affinity of wild-type ATG4B and its mutants with UrA.

(F) A table summarizing molecular interaction studies of the different ATG4B protein mutants.

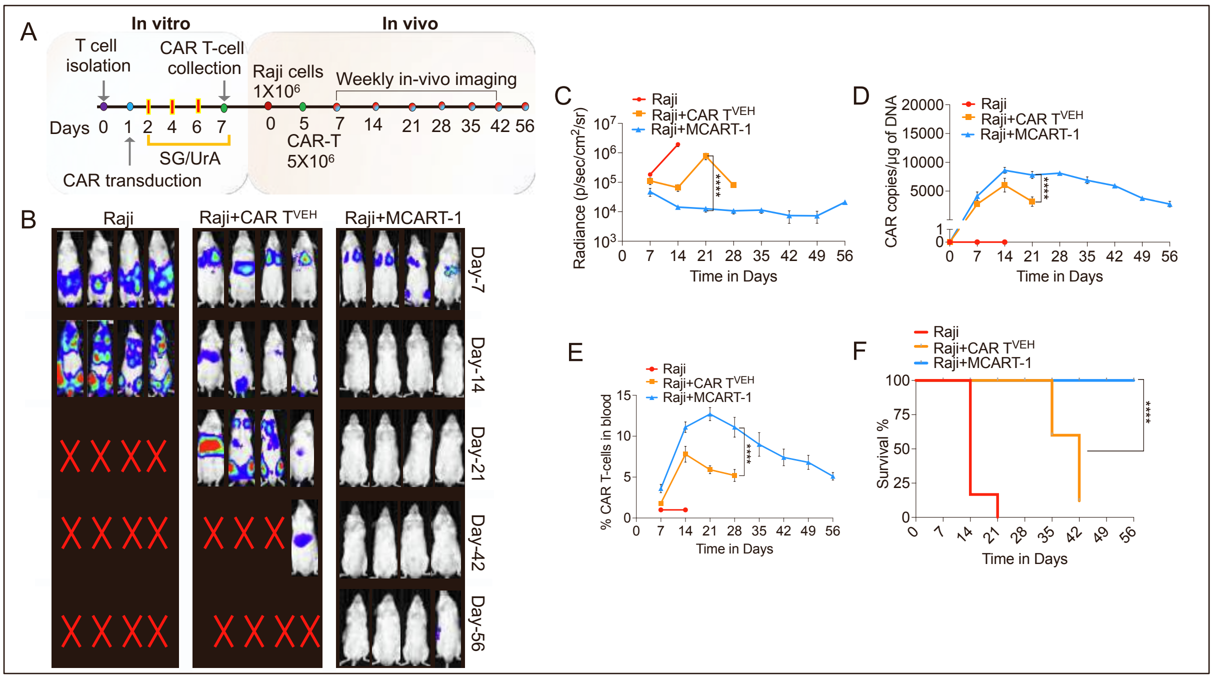

Figure S20: Low dose of MCAR T-1 exhibits robust anti-tumor activity and persistence as compared to conventional CAR T cells.

(A) Schematic of the Raji tumor model, with similar isolation, transduction, and treatment of CAR T cells with either VEH or SG + UrA) and administered to mice. Raji cells were injected into mice, followed by CAR T cell infusion (5×10^6 cells) on day 5. In vivo imaging was performed weekly from day 7 to day 56.

(B) Images showing tumor burden in mice treated over time (days 7, 14 and 28) (n=4).

(C) Quantification of bioluminescence radiance (photons/sec/cm²/sr) in Raji tumor-bearing mice treated with CAR T cells over time (n=5).

(D, E) CAR transgene and CAR T cells detected in the blood over time (n=5).

(F) Survival plots of the mice over time of 56 days (n=5).

Raji group blood cell analysis was done on day 7, 14 and 20. Data represents mean ± SE; from three independent experiments. *$p < 0.05$. A non-parametric t-test was used for statistical analysis. The Mantel-Cox test was done to compare survival between two groups.

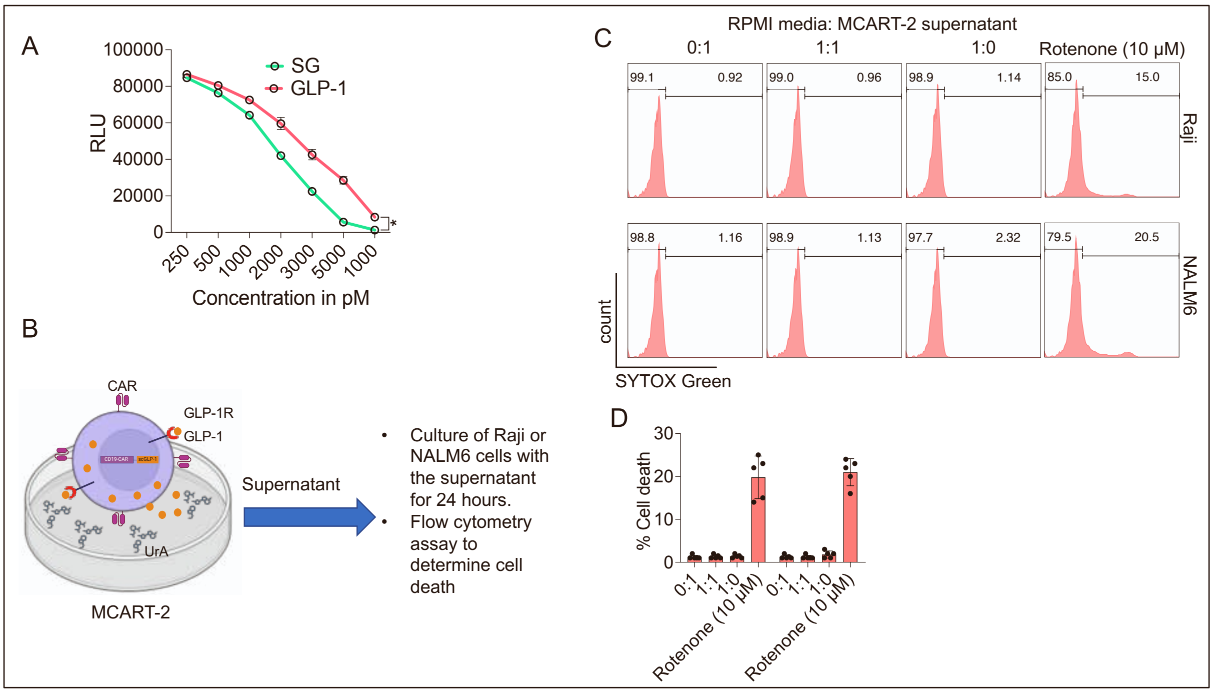

Figure S21: Evaluation of nGLP-1 secretion and anti-tumor effects of MCAR T-2 cells.

(A) Comparison of cAMP activation levels induced by nGLP-1 and SG in a stable GLP-1R-expressing CHO cell line (n=5). The data is represented as relative light unit (RLU).

(B) Schematic of scGLP-1/CAR T cell functionality showing the secretion of GLP-1. Supernatant from scGLP-1/CAR T cells was collected and incubated with Raji or NALM6 cells for 24 hours to assess any direct cytotoxicity.

(C-D) Flow cytometry analysis of Raji and NALM6 cells cultured with scGLP-1/CAR T cell supernatant. Minimal cell death was observed compared to the positive control (Rotenone 10 μM), suggesting that scGLP-1 and UrA act through enhancing CAR T cell signaling rather than directly inducing tumor cell death (n=5).

Data represents mean ± SE; from three independent experiments. $*p < 0.05$. A non-parametric t-test was used for statistical analysis.

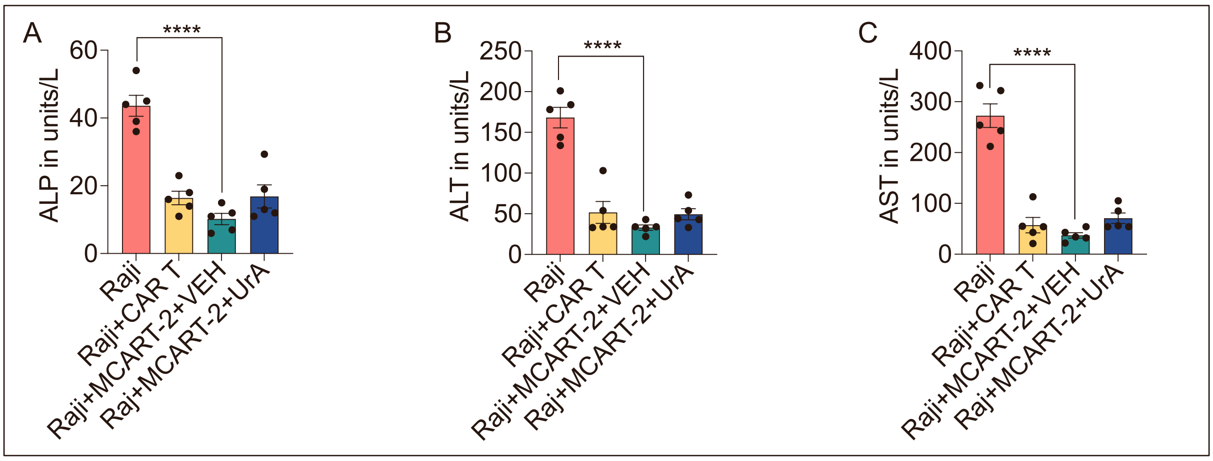

Figure S22: Tissue toxicity analysis in mice treated with MCAR T-2 cells

(A-C) Serum levels of tissue toxicity markers, including alkaline phosphatase (ALP) (A), alanine transaminase (ALT) (B), and aspartate transaminase (AST) (C), were measured to assess liver function and potential tissue toxicity.

Data represents mean ± SE; from three independent experiments. ****p < 0.0001. A non-parametric t-test was used for statistical analysis.

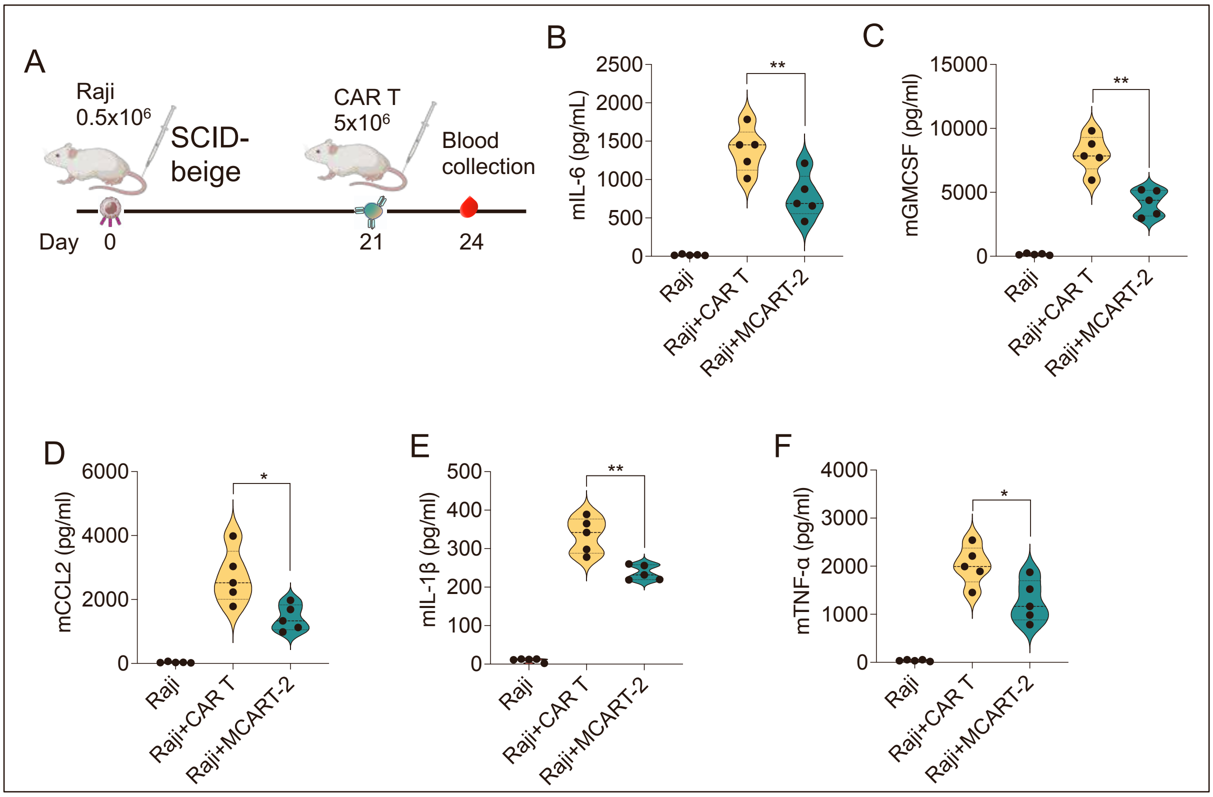

Figure S23: MCAR T-2 cells show lower CRS in mice model:

(A) Shows the general schematics of the CRS mice model development using 4–6 week old *C.B.Igh-1b/GbmsTac-PrkdcscidLystbgN7* (SCID-beige) mice. (B-F) Measurement of the cytokines in the sera obtained

from peripheral blood of the mice at day 24 (n=5). Data represents mean ± SE; from three independent experiments. $^{**}p < 0.01$; $^{*}p < 0.05$; ns (not significant). A non-parametric t-test was used for statistical analysis.
